# Expected KL risk quantifies when first-order power-law approximations are sufficient

**DOI:** 10.1038/s41598-026-55101-y

**Published:** 2026-05-27

**Authors:** Chikoo Oosawa

**Affiliations:** Department of Physics and Information Technology, Kyushu Institute of Technology, 680–4 Kawazu, Iizuka, 820-8502 Fukuoka Japan

**Keywords:** Biochemical systems theory, Log-synergism, Information geometry, Kullback–Leibler risk, Approximation error, Model reduction, Mathematics and computing, Physics

## Abstract

Biochemical Systems Theory (BST) often replaces nonlinear rate laws by first-order log–Taylor power-law approximations, but deciding when this truncation is adequate remains difficult. We derive a closed-form leading-order expression for the expected conditional Kullback–Leibler (KL) risk incurred by using the first-order model instead of the local second-order log expansion. Under Gaussian log-input fluctuations with covariance *Σ* and homoscedastic Gaussian log-output noise with variance $s^2$, the risk reduces to a trace contraction of the local log-curvature Hessian *H* with *Σ*. The criterion is therefore directly estimable from perturbation data or mechanistic models near an operating point. We also identify the leading correction from non-Gaussian inputs through fourth-order cumulants. Toy-model calculations and two biochemical case studies show that the criterion not only matches Monte Carlo estimates, but also identifies operating conditions and perturbation directions for which first-order BST is expected to fail.

## Introduction

Power-law representations and log-linearizations are ubiquitous in the analysis of biochemical networks^1,2^. In BST, a nonlinear rate law $v(\boldsymbol{x})$ is locally approximated near a reference state $\boldsymbol{x}^*$ by a power-law form with exponents given by log-gains. This approximation is extremely convenient for sensitivity analysis, design comparison, and reduced modeling; it also underpins the analytical tractability of the S-system formalism. However, the log response *łog v* generally has nonzero curvature in log-coordinates, and in particular it contains mixed second derivatives that encode non-multiplicative interaction between inputs. Salvador introduced *log-synergism* to systematize the computation and interpretation of these second-order interaction terms^3,4^.

The present work asks a quantitative question that is natural from an information-theoretic viewpoint: *What is the cost of using a first-order (multiplicative) BST model when the true system exhibits log-synergism?* In statistical terms, this is the excess predictive loss when one uses an approximate distribution *q* in place of the true distribution *p*; it is measured by $D_{\textrm{KL}}(p\Vert q)$. Our main contribution is a closed-form approximation for the *expected* KL risk under small log-fluctuations, expressed directly in terms of the log-synergism Hessian *H* and the input covariance *Σ*, which can be used as a practical diagnostic for when higher-order interaction terms become necessary. Related KL divergences between forward and time-reversed *path* measures also appear in nonequilibrium thermodynamics as measures of irreversibility and dissipation^5–7^; for optional context we provide a brief note in the Supplementary Information.

Seen more broadly, the BST1–BST2 distinction admits a local geometric reading in the space of probabilistic models. Near a reference operating point, the KL divergence between nearby distributions is well approximated by a quadratic form determined by the Fisher information metric, so the first-order log–Taylor (power-law) approximation can be interpreted as locally “flattening” the model manifold, whereas the log-synergism Hessian provides the leading curvature correction^8,9^. Viewing the BST1 reduction as a local coarse-graining also makes the directionality of model error transparent through the data-processing inequality^10^.

Practically, the same closed-form expected KL risk provides an operating-point criterion for when a first-order multiplicative approximation is sufficient, and it also informs perturbation design: because the risk scales with contractions of *H* against the input covariance *Σ*, conditions that amplify fluctuations along strongly curved directions are predicted to reveal the failure of the first-order model most clearly.

More broadly, BST’s operating-point (steady-state) viewpoint is closely related to the steady-state analysis used across dynamical systems and control engineering. For a general state-space system $\dot{\boldsymbol{x}}=f(\boldsymbol{x},\boldsymbol{u})$ with output $\boldsymbol{y}=g(\boldsymbol{x},\boldsymbol{u})$, a steady state $(\boldsymbol{x}^*,\boldsymbol{u}^*)$ satisfies $f(\boldsymbol{x}^*,\boldsymbol{u}^*)=\boldsymbol{0}$, and linearization about this point yields static (DC) gains/sensitivities for constant perturbations (e.g. $\delta \boldsymbol{y}_{\textrm{ss}}=(D-CA^{-1}B)\,\delta \boldsymbol{u}$ in standard notation). BST can be viewed as a normalized version of this operating-point linearization carried out in log-coordinates: the log-gains in Eq. (2) are dimensionless incremental gains (elasticities). In this work, we further complement the usual first-order operating-point approximation by providing an explicit KL-risk criterion, Eq. (10), that quantifies when curvature (log-synergism) makes the first-order approximation information-theoretically costly^11,12^.

### Contributions

We make four concrete contributions. First, we recast the gap between a first-order BST approximation (BST1) and its local second-order log expansion (BST2) as an expected conditional KL risk. Second, under Gaussian log-input fluctuations with covariance *Σ* and homoscedastic Gaussian log-output noise with variance $s^2$, we derive the closed-form leading-order risk in Eq. (10). Third, we make the scope of this result explicit by separating the leading non-Gaussian correction, which enters through fourth-order cumulants. Fourth, we turn the result into an operational decision rule: at a chosen operating point, BST1 is declared sufficient when the estimated risk remains below a user-specified tolerance $\tau _{\textrm{KL}}$, or conservatively when the upper uncertainty bound stays below $\tau _{\textrm{KL}}$. In practice, $\tau _{\textrm{KL}}$ is application-dependent and may be chosen relative to tolerated excess predictive loss, measurement resolution, or downstream decision risk.

### Relation to local steady-state frameworks

Our criterion complements local analyses around a steady state, such as structural kinetic modeling (SKM) and modular response analysis (MRA), which parameterize or infer elasticities/response coefficients from models or perturbation data. In contrast, we quantify the *information-theoretic cost* of truncating nonlinear kinetics at first order in log-coordinates and use it for model selection and perturbation design^13,14^.

The result is intentionally local: it assumes a valid second-order log expansion in a neighborhood of the operating point, a conditional observation model for the log-output, and small enough fluctuations that higher-than-quadratic terms are negligible at leading order. These assumptions are discussed explicitly below and in the Supplementary Information, because the purpose of the criterion is not to replace full mechanistic analysis globally, but to diagnose when a first-order local approximation is adequate.

## BST first-order log–Taylor approximation

Let $u_j=\log x_j$ denote log-coordinates. A first-order log–Taylor expansion around $\boldsymbol{u}^*=\log \boldsymbol{x}^*$ gives

1 $$\begin{aligned} \log v(\boldsymbol{u})=\log v(\boldsymbol{u}^*)+\sum _j g_j (u_j-u_j^*)+\mathcal {O}(\Vert \boldsymbol{u}-\boldsymbol{u}^*\Vert ^2), \end{aligned}$$

with log-gains

2 $$\begin{aligned} g_j=\left. \frac{\partial \log v}{\partial \log x_j}\right| _{\boldsymbol{x}^*}. \end{aligned}$$

Exponentiating Eq. (1) yields the BST power-law approximation

3 $$\begin{aligned} v(\boldsymbol{x})\approx v(\boldsymbol{x}^*)\prod _{j}\left( \frac{x_j}{x_j^*}\right) ^{g_j}. \end{aligned}$$

This is the basic local building block behind S-systems, e.g.

4 $$\begin{aligned} \frac{d x_i}{dt}=\alpha _i\prod _j x_j^{g_{ij}}-\beta _i\prod _j x_j^{h_{ij}}. \end{aligned}$$

## Log-synergism as curvature in log-coordinates

The curvature discarded by the first-order approximation is captured by the Hessian of the log response,

5 $$\begin{aligned} H_{jk}:=\left. \frac{\partial ^2 \log v}{\partial u_j\,\partial u_k}\right| _{\boldsymbol{u}^*} =\left. \frac{\partial ^2 \log v}{\partial \log x_j\,\partial \log x_k}\right| _{\boldsymbol{x}^*}. \end{aligned}$$

Salvador’s log-synergism can be defined as a finite-difference measure that vanishes for strictly multiplicative responses, and Eq. (5) provides its differential approximation^3^. Mixed components $H_{jk}$ (*j≠ k*) quantify non-multiplicative interaction between inputs in log-coordinates.

## Information-geometric motivation

### Information-geometric view of the first-order approximation

Let *u=łog x* denote log-coordinates for the inputs *x*. The first-order log–Taylor approximation replaces a nonlinear log-response *łog v(u)* by its local affine expansion, $\log v(u)\simeq \log v(u^*) + g^\top (u-u^*)$. In information-geometric terms, restricting to such log-affine (multiplicative) response families corresponds to a locally “flat” model class in the exponential (e-) connection, i.e. an *e-affine* submanifold whose natural parameters depend affinely on *u*^8^. From this viewpoint, Salvador’s log-synergism (the mixed second derivatives of *łog v* in *u*) is the local curvature object that controls the leading-order deviation from multiplicativity and therefore the KL-risk expansion derived below.

We consider the conditional observation models

6 $$\begin{aligned} p(z|\boldsymbol{u})=\mathcal {N}\!\left( z;\mu (\boldsymbol{u}),s^2\right) ,\qquad q(z|\boldsymbol{u})=\mathcal {N}\!\left( z;\mu _{\textrm{BST1}}(\boldsymbol{u}),s^2\right) , \end{aligned}$$

where *z* is the observed log-output and both models share the same observation-noise variance $s^2$. The conditional Kullback–Leibler divergence is defined by

7 $$\begin{aligned} D_{\textrm{KL}}\!\bigl (p(\cdot |\boldsymbol{u})\Vert q(\cdot |\boldsymbol{u})\bigr ):= \int p(z|\boldsymbol{u})\log \!\frac{p(z|\boldsymbol{u})}{q(z|\boldsymbol{u})}\,\textrm{d}z . \end{aligned}$$

Because Eq. (6) uses equal-variance Gaussians, the variance terms cancel exactly and Eq. (7) reduces to

8 $$\begin{aligned} D_{\textrm{KL}}\!\bigl (p(\cdot |\boldsymbol{u})\Vert q(\cdot |\boldsymbol{u})\bigr )= \frac{\bigl (\mu (\boldsymbol{u})-\mu _{\textrm{BST1}}(\boldsymbol{u})\bigr )^2}{2s^2}. \end{aligned}$$

A short derivation is given in the Supplementary Information section “KL divergence between equal-variance Gaussians”.

### Closed form under Gaussian input fluctuations

Let $\delta \boldsymbol{u}=\boldsymbol{u}-\boldsymbol{u}^*$ and assume $\delta \boldsymbol{u}\sim \mathcal {N}(\boldsymbol{0},\Sigma )$. The local mean mismatch is

9 $$\begin{aligned} \Delta \mu (\boldsymbol{u}):=\mu (\boldsymbol{u})-\mu _{\textrm{BST1}}(\boldsymbol{u}) =\frac{1}{2}\delta \boldsymbol{u}^\top H\,\delta \boldsymbol{u}+\mathcal {O}(\Vert \delta \boldsymbol{u}\Vert ^3). \end{aligned}$$

Substituting Eq. (9) into Eq. (8) gives

$$D_{\textrm{KL}}(p\Vert q)=\frac{1}{8s^2}\bigl (\delta \boldsymbol{u}^\top H\,\delta \boldsymbol{u}\bigr )^2+\mathcal {O}(\Vert \delta \boldsymbol{u}\Vert ^5).$$

Taking the expectation over $\delta \boldsymbol{u}$ and using the Gaussian fourth-moment identity (Wick/Isserlis; see the Supplementary Information section entitled “Gaussian fourth-moment identity (Wick/Isserlis)”) yields

10 $$\begin{aligned} \mathbb {E}_{\delta \boldsymbol{u}}\!\left[ D_{\textrm{KL}}(p\Vert q)\right] = \frac{1}{8s^2}\Bigl \{2\,\textrm{tr}\!\bigl [(H\Sigma )^2\bigr ]+\bigl (\textrm{tr}(H\Sigma )\bigr )^2\Bigr \} +\mathcal {O}\!\left( \Vert \Sigma \Vert ^{5/2}\right) . \end{aligned}$$

Equation (10) makes the logic of the reduction explicit: Eq. (7) becomes Eq. (8) because the two conditional models share the same Gaussian variance, and Eq. (8) becomes Eq. (10) because the leading mismatch is quadratic in $\delta \boldsymbol{u}$ and its square has a closed Gaussian fourth moment. Even if inputs are uncorrelated (*Σ* diagonal), mixed terms $H_{jk}$ contribute through $\textrm{tr}[(H\Sigma )^2]$. Unlike Monte Carlo agreement alone, the closed form separates curvature-, covariance-, and noise-scale effects, identifies high-risk perturbation directions before simulation, and can be estimated directly from local perturbation data.

## Numerical demonstration (toy model)

**Fig. 1:**
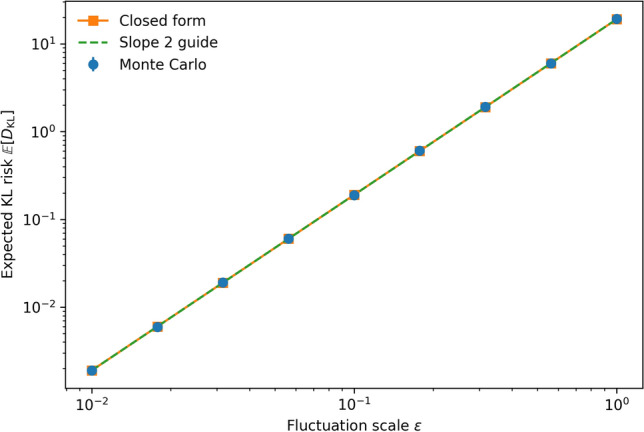
Comparison of the expected KL risk $\mathbb {E}[D_{\textrm{KL}}(p\Vert q)]$ between the closed-form prediction, Eq. (10), and Monte Carlo estimation for the toy model in Eq. (11). Error bars show the standard error of the Monte Carlo mean.

To verify Eq. (10), we consider a two-dimensional toy model with

11 $$\begin{aligned} \mu (\boldsymbol{u})=\mu _0+\boldsymbol{g}^\top \boldsymbol{u}+\frac{1}{2}\boldsymbol{u}^\top H\boldsymbol{u}, \quad H=\begin{pmatrix}1.2& -0.7 \\ -0.7& 0.9\end{pmatrix}, \end{aligned}$$

and *s=0.2*. We draw $\boldsymbol{u}\sim \mathcal {N}(\boldsymbol{0},\Sigma )$ with $\Sigma =\varepsilon \,\Sigma _0$ for $$\Sigma _0=\begin{pmatrix}1.0& 0.6\\ 0.6& 1.5\end{pmatrix}$$, and estimate $\mathbb {E}[D_{\textrm{KL}}]$ by Monte Carlo. Figure 1 shows excellent agreement between the closed-form prediction, Eq. (10), and Monte Carlo estimates across a wide range of *\varepsilon*, together with the expected scaling $\mathbb {E}[D_{\textrm{KL}}]\propto \varepsilon ^2$ (log–log slope *≈ 2*). For reproducibility, the figure was generated with $N=2\times 10^5$ samples per point and a fixed random seed.

## Case study: two-substrate enzyme kinetics (ordered Bi–Bi)

As a compact biochemical example with an explicit mechanistic rate law and strictly positive inputs, we consider an ordered Bi–Bi (two-substrate) enzyme mechanism in the sense of Cleland’s nomenclature^15^. Let *A* and *B* denote substrate concentrations. A convenient representative rate law is

12 $$\begin{aligned} v(A,B)=V_{\max }\,f_{\textrm{allo}}(E)\,\frac{AB}{K_{\textrm{ia}}K_{\textrm{b}}\,\bigl (1+I/K_{\textrm{i}}\bigr )+K_{\textrm{a}}B+K_{\textrm{b}}A+AB}, \end{aligned}$$

where $K_{\textrm{a}},K_{\textrm{b}}$ are Michaelis constants, $K_{\textrm{ia}}$ is the dissociation constant for the first substrate in the ordered mechanism, and *I* denotes a competitive inhibitor (for definiteness, modeled here as a multiplicative factor on the $K_{\textrm{ia}}K_{\textrm{b}}$ term). To keep the example minimal yet realistic, we also include an allosteric modulation of $V_{\max }$ via $f_{\textrm{allo}}(E)=1+\gamma \,\frac{E}{K_E+E}$, motivated by standard coarse-grained allosteric response functions^16,17^.

We choose an operating point $(A^*,B^*)$ and define the log response $\mu (\boldsymbol{u})=\log v(A^*e^{u_1},B^*e^{u_2})$ with $\boldsymbol{u}=(u_1,u_2)$. Using the finite-difference recipe in Methods, we estimate the local log-gains $\boldsymbol{g}$ and the log-curvature Hessian *H* at $\boldsymbol{u}=\boldsymbol{0}$. For the parameter set in Table 1 (dimensionless scaling; *s=0.2*), we obtain $H_{12}\approx -0.213$, comparable in magnitude to $H_{11}\approx H_{22}\approx -0.249$. Thus the mixed curvature (log-synergism) is not negligible at this operating condition.

**Table 1:** Parameters for the ordered Bi–Bi enzyme-kinetics case study (Eq. 12).

Parameter	Value	Meaning
$V_{\max }$	1.0	Maximal rate (scaled)
$K_{\textrm{a}}$	0.086	Michaelis constant for *A* (scaled)
$K_{\textrm{b}}$	0.110	Michaelis constant for *B* (scaled)
$K_{\textrm{ia}}$	9.60	Dissociation constant for ordered binding
*I*	0.50	Ihibitor level (fixed)
$K_{\textrm{i}}$	1.00	Inhibition constant (scaled)
*E*	1.00	Effector level (fixed)
*γ*	1.00	Allosteric strength
$K_E$	1.00	Allosteric half-saturation
*s*	0.20	Log-output noise SD

Substrates are scaled so that the operating point is $(A^*,B^*)=(1.0,1.35)$.

**Fig. 2:**
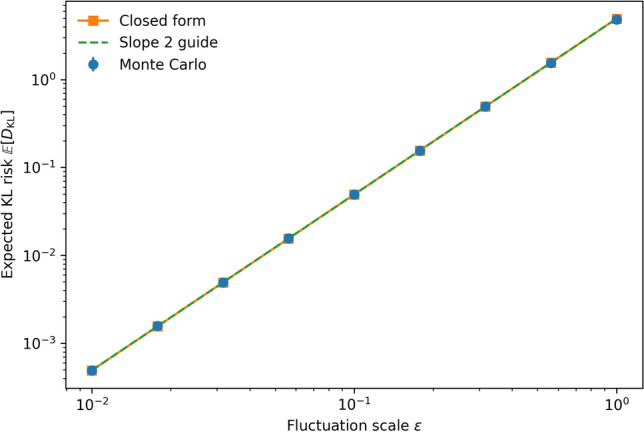
Ordered Bi–Bi enzyme-kinetics case study (Eq. 12). Comparison of the predicted expected KL risk (closed form, Eq. 10) with Monte Carlo estimation under log-Gaussian substrate fluctuations $\Sigma =\epsilon \Sigma _0$. Error bars show the standard error of the Monte Carlo mean.

We next test the predictive content of Eq. (10) for this mechanistic rate law. We sample log-input fluctuations $\delta \boldsymbol{u}\sim \mathcal {N}(\boldsymbol{0},\Sigma )$ with $\Sigma =\epsilon \,\Sigma _0$ and $$\Sigma _0=\begin{pmatrix}1.0& 0.4\\ 0.4& 1.3\end{pmatrix}$$. Figure 2 shows that the closed-form prediction in Eq. (10) matches Monte Carlo estimates of $\mathbb {E}[D_{\textrm{KL}}]$ across a wide range of fluctuation scales *ε*. The expected quadratic scaling $\mathbb {E}[D_{\textrm{KL}}]\propto \epsilon ^2$ is also observed.

**Fig. 3:**
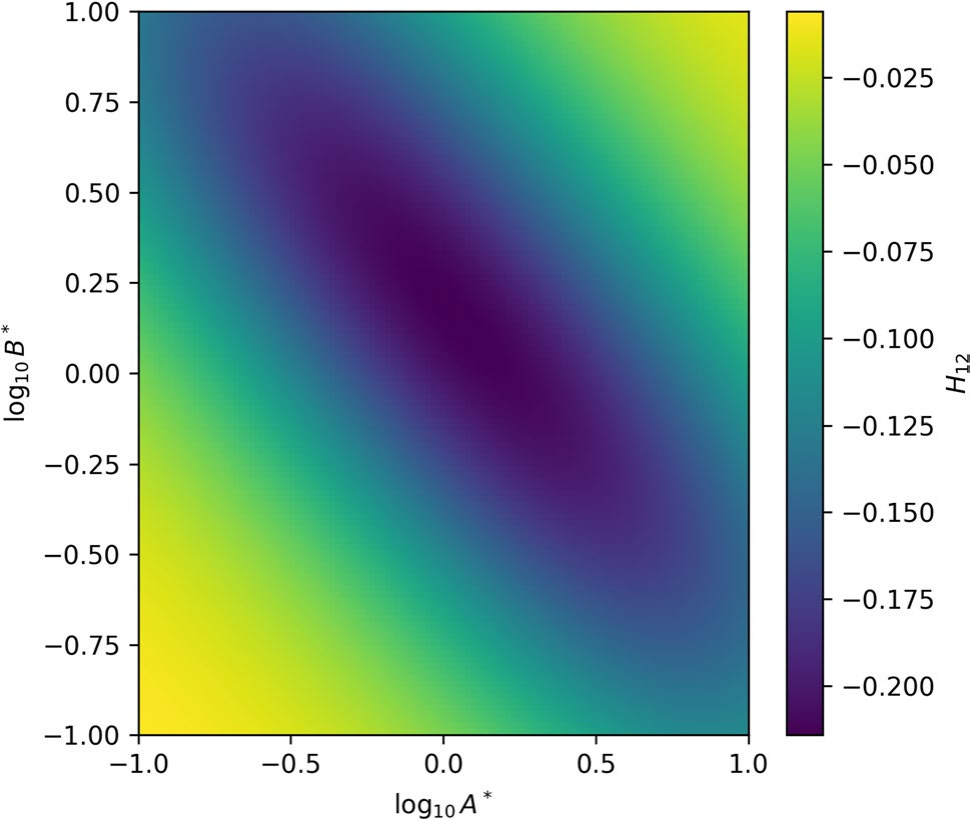
Operating-point dependence of the mixed log-curvature (log-synergism) term $H_{12}$ for the ordered Bi–Bi rate law (Eq. 12) with other parameters fixed as in Table 1. Axes show $\log _{10}A^*$ and $\log _{10}B^*$; the color encodes $H_{12}$.

Finally, to emphasize that log-synergism is operating-point dependent even for a fixed mechanism, Fig. 3 maps the mixed curvature $H_{12}$ over a range of operating points $(A^*,B^*)$. Such maps identify experimental regimes where interaction terms are expected to matter most and therefore show the practical value of the criterion beyond agreement with Monte Carlo alone: the same mechanistic model can be locally safe for BST1 at some operating points and clearly unsafe at others.

## Case study: thermodynamic gene regulation (activator–repressor competition)

As a second biochemical illustration beyond enzyme kinetics, we consider a minimal two-input transcriptional response motivated by thermodynamic promoter-occupancy models of gene regulation^18,19^. Let *A* and *R* denote concentrations of an activator and a repressor, respectively. A compact phenomenological form that captures competitive binding and a tunable interaction between inputs is

13 $$\begin{aligned} v(A,R)=V_{\max }\, \frac{1+a\,(A/K_A)^n}{1+(A/K_A)^n+(R/K_R)^m+\omega \,(A/K_A)^n(R/K_R)^m}, \end{aligned}$$

where *n*, *m* are Hill exponents, *a* sets the strength of activation, and *ω* controls how strongly the joint (*A* and *R*) occupancy state contributes to the partition function. When $\omega \ne 0$, the log-response $\mu =\log v$ is not additively separable in *łog A* and *łog R*, and the mixed log-curvature $H_{12}$ becomes nonzero.

**Fig. 4:**
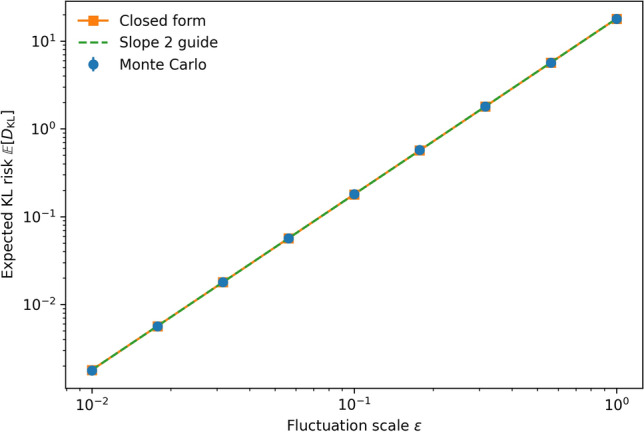
Thermodynamic gene-regulation case study (Eq. 13). Comparison of the predicted expected KL risk (closed form, Eq. (10)) with a direct Monte Carlo estimate of Eq. (8), for log-Gaussian input fluctuations $\Sigma =\epsilon \Sigma _0$ (see text). The dashed line is a slope-2 guide. Error bars show the standard error of the Monte Carlo mean.

We choose an operating point $(A^*,R^*)=(K_A,K_R)$ with parameters $V_{\max }=1$, *a=8*, *n=m=2*, *ω =3*, and take *s=0.2* in Eq. (6). Estimating *H* at $\boldsymbol{u}=\boldsymbol{0}$ (Methods) gives $H_{12}\approx -0.222$, with diagonal curvatures $H_{11}\approx -0.494$ and $H_{22}\approx -0.889$. Figure 4 shows that the closed-form risk (Eq. 10) accurately predicts the Monte Carlo estimate over a decade of fluctuation scales for log-Gaussian inputs $\Sigma =\epsilon \Sigma _0$ with $$\Sigma _0=\begin{pmatrix}1 & 0.35\\ 0.35 & 1\end{pmatrix}.$$ This example illustrates how the same diagnostic applies to a broad class of mechanistic or phenomenological rate laws (including models curated in BioModels^20^), where mixed input interactions generate failure modes of multiplicative approximations. In particular, the criterion distinguishes low-risk from high-risk fluctuation designs even when Monte Carlo verification is not yet available, which is the practical setting in which a local diagnostic is most useful.

## Discussion

**Fig. 5:**
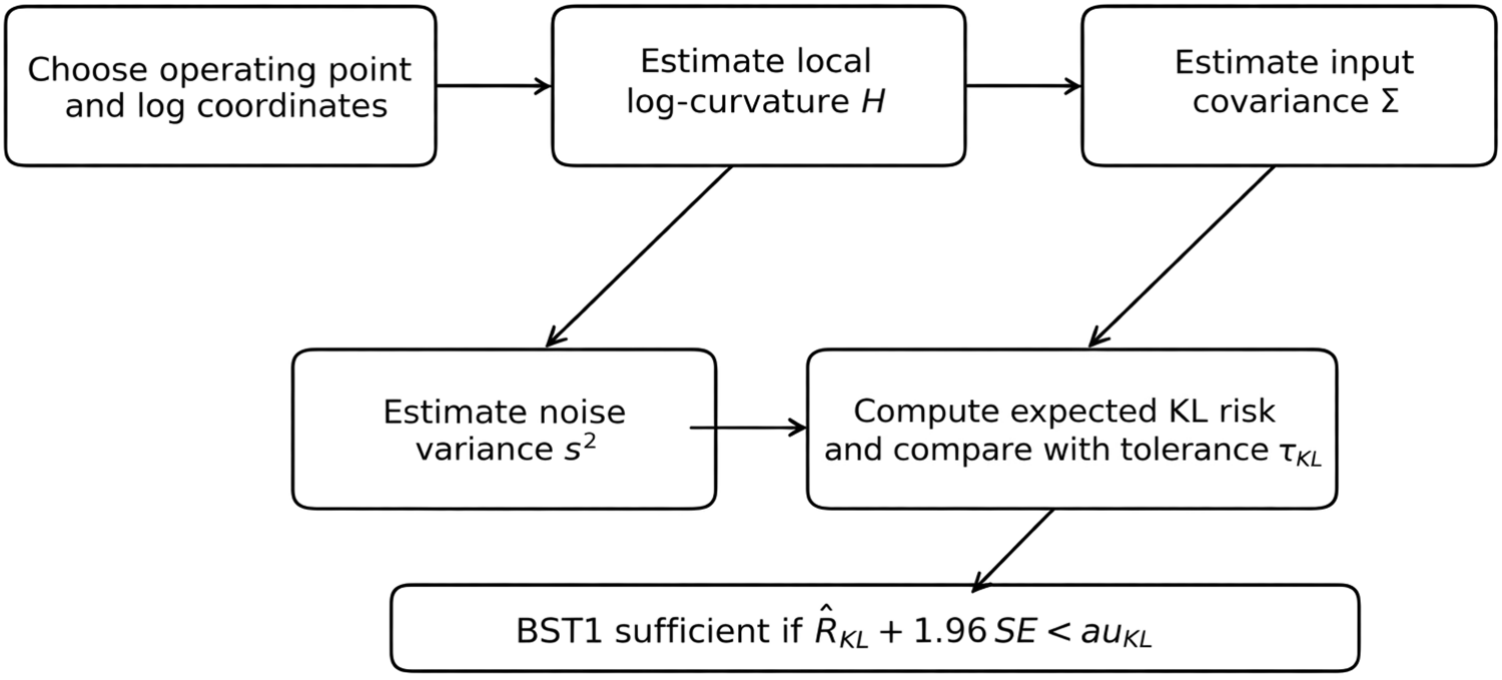
A practical recipe for estimating the expected KL risk of a first-order BST approximation. Starting from a model or perturbation data near an operating point $\boldsymbol{x}^*$, estimate the log-curvature *H* (log-synergism Hessian) and the fluctuation covariance *Σ* (and a noise scale $s^2$), then evaluate Eq. (10) to decide whether interaction terms are negligible or warrant explicit modeling.

A practical use of Eq. (10) is as a *diagnostic* for when first-order (multiplicative) BST models are expected to be adequate. Figure 5 and Algorithm 1 summarize a minimal workflow from data (or a mechanistic rate law) to a predicted excess loss. We provide additional robustness checks in the Supplementary Information (Supplementary Figs. S1–S4), illustrating (i) the effect of heavy-tailed log-fluctuations with matched covariance, (ii) robustness under general (correlated) covariance structure, (iii) the expected $1/s^2$ dependence on noise level, and (iv) how mechanistic choices can alter mixed log-curvature and the predicted risk.

Equation (10) provides a simple diagnostic: estimate the local log-synergism Hessian *H* (e.g. via a quadratic fit of $\mu (\boldsymbol{u})$) and the fluctuation covariance *Σ* from data, and then predict the leading-order information loss of adopting a first-order BST approximation. This suggests an operational criterion for when higher-order interaction terms are *required* to avoid large information-theoretic penalties.

A key reason BST is broadly useful is that its “steady-state” viewpoint is closely homologous to the operating-point analysis that is standard in dynamical systems, circuit theory, and control engineering. In a general state-space model $\dot{\boldsymbol{x}}=f(\boldsymbol{x},\boldsymbol{u})$, a steady state (equilibrium/operating point) is a pair $(\boldsymbol{x}^*,\boldsymbol{u}^*)$ satisfying $f(\boldsymbol{x}^*,\boldsymbol{u}^*)=\boldsymbol{0}$, and small-signal behavior is obtained by linearizing the dynamics about this point to compute a Jacobian $A=\partial f/\partial \boldsymbol{x}$ and an input matrix $B=\partial f/\partial \boldsymbol{u}$. For stable equilibria, the resulting linear model yields static (DC) gains and steady-state sensitivities, for instance $\delta \boldsymbol{x}_{\textrm{ss}}=-A^{-1}B\,\delta \boldsymbol{u}$ and $\delta \boldsymbol{y}_{\textrm{ss}}=(D-CA^{-1}B)\,\delta \boldsymbol{u}$ in the standard notation^11,12^. BST can be viewed as a particular *normalized* operating-point linearization carried out in log-coordinates: the log-gains $g_j=\partial \log v/\partial \log x_j|_{\boldsymbol{x}^*}$ are dimensionless incremental gains (elasticities), and converting back to physical units gives $\partial v/\partial x_j=(v^*/x_j^*)\,g_j$. Moreover, for S-systems the steady-state conditions become linear equations in the log-variables $\log x_i^*$, which enables closed-form expressions for steady-state values and static sensitivities—a direct analogue of DC operating-point computation and sensitivity analysis in control and circuit settings.

From this perspective, Eq. (10) provides something that classical linearization typically does not: a quantitative, data-driven *error measure* for the first-order operating-point approximation. It specifies how the neglected second-order interaction structure (log-synergism curvature *H*) couples with fluctuation statistics *Σ* to produce an excess predictive loss, thereby clarifying when a first-order power-law (BST1) description is sufficient and when higher-order interaction terms are information-theoretically unavoidable.

Beyond the static setting of Eq. (10), the same KL-risk perspective can be used to formalize and compare *numerical* reductions of nonlinear biochemical dynamics. For instance, discrete formulations of BST (“dBST”) represent S-system-type dynamics as explicit update maps with a user-chosen step size and, in practice, solver and tolerance choices when continuous-time ODE models are used instead^21^. In such contexts, neglected interaction structure (captured here by *H*) may couple with discretization artifacts and input fluctuations to yield an additional, operationally meaningful information loss. This observation suggests a concrete future direction: choose discretization parameters (e.g. step size, integration scheme, tolerances) by minimizing an empirically estimated KL risk relative to a high-accuracy reference, or by using data-estimated *(H,Σ )* to predict regimes where low-order approximations are likely to be unreliable.

More broadly, the same risk can be used in several complementary ways: (i) to trigger adaptive refinement when the predicted loss exceeds a chosen threshold; (ii) to decide when to switch from a first-order (BST1) description to higher-order or hybrid approximations; (iii) to regularize dBST parameter estimation toward parsimonious interaction structure; and (iv) to guide perturbation and measurement design by prioritizing fluctuation directions that most strongly probe mixed log-curvature.

Another natural extension is to integrate the present information-theoretic diagnostic into the methodology of *mathematically controlled comparison* (MCC), which was explicitly extended to include numerical comparisons across model classes^22^. In the MCC spirit, one may treat the expected KL risk (or related information-theoretic costs) as a “systemic property” to be compared across competing modeling choices, thereby providing a unified criterion that is both statistically interpretable (excess predictive loss). Within mathematically controlled comparison (MCC) studies, the KL-based risk can likewise play multiple roles: (i) a pre-screening fairness criterion that restricts comparisons to regimes where competing designs are approximated with comparable risk; (ii) an additional outcome variable reported alongside fluxes, concentrations, and gains to expose trade-offs between functional performance and approximability; (iii) an explicit matching constraint that equalizes the information cost of coarse graining across designs; (iv) a decomposition tool that attributes observed differences to specific interaction curvatures and fluctuation directions; (v) a way to design the comparison domain by mapping where the risk is high or low; and (vi) a multi-objective selection principle that balances functional effectiveness against information loss.

## Conclusion

We have recast the difference between BST first-order log–Taylor (power-law) approximations and local second-order log curvature in an information-theoretic language. Under the stated local-expansion and Gaussian-observation assumptions, the expected KL risk of using a first-order BST model admits the closed-form expression in Eq. (10), controlled by contractions of the log-synergism Hessian with the input covariance. This turns the adequacy of BST1 into an explicit, data-driven model-selection problem and provides a practical bridge from classical biochemical sensitivity constructs to quantitative predictive loss. The present criterion is local and leading-order: it assumes a valid second-order log expansion around the operating point and equal-variance Gaussian observation noise, so extensions to broader operating domains and heteroscedastic or strongly non-Gaussian observation models remain future work. A brief optional discussion of trajectory-level information-thermodynamic connections is provided in the Supplementary Information^6,23^.

## Methods

### Practical workflow (overview)

Figure 5 summarizes a minimal, data-to-decision workflow for using the main bound, Eq. (10), as a diagnostic: it links a local estimate of log-curvature (*H*), a characterization of input fluctuations (*Σ*), and an observation noise scale ($s^2$) to a predicted excess loss $\mathbb {E}[D_{\textrm{KL}}(p\Vert q)]$ incurred by a first-order (multiplicative) BST approximation.

*Algorithm 1 (minimal practical recipe)*

1. Choose an operating point $\boldsymbol{x}^*$ (e.g. steady state, typical condition) and define log-coordinates $\boldsymbol{u}=\log \boldsymbol{x}$.
2. Estimate the local log-gains $\boldsymbol{g}=\nabla _{\boldsymbol{u}}\mu (\boldsymbol{u}^*)$ and the log-curvature $H=\nabla _{\boldsymbol{u}}^2\mu (\boldsymbol{u}^*)$ of $\mu (\boldsymbol{u})=\log v(\boldsymbol{u})$ via a quadratic fit or finite differences.
3. Estimate the covariance $\Sigma =\textrm{Cov}(\delta \boldsymbol{u})$ of log-input fluctuations $\delta \boldsymbol{u}=\boldsymbol{u}-\boldsymbol{u}^*$ from perturbation data (or adopt a design prior, e.g. $\Sigma =\epsilon \,\Sigma _0$).
4. Specify an observation noise variance $s^2$ for the (log-)output (from replicates or a measurement model).
5. Compute the predicted excess loss $\widehat{R}_{\textrm{KL}}:=\mathbb {E}[D_{\textrm{KL}}(p\Vert q)]$ using Eq. (10). Choose a domain-specific tolerance $\tau _{\textrm{KL}}$ and declare BST1 sufficient only if $\widehat{R}_{\textrm{KL}}<\tau _{\textrm{KL}}$ (or conservatively if $\widehat{R}_{\textrm{KL}}+1.96\,\textrm{SE}<\tau _{\textrm{KL}}$); otherwise, include interaction terms or refine experiments to resolve the dominant entries of *H*. In practice, $\tau _{\textrm{KL}}$ should be calibrated to tolerated excess predictive loss, measurement resolution, or downstream decision risk.

*Checklist for experimental estimation (quick start)*

1. Define the operating point and log-variables. Choose $\boldsymbol{x}^*$ and compute $\boldsymbol{u}=\log \boldsymbol{x}$, ensuring strictly positive inputs; center perturbations as $\delta \boldsymbol{u}=\boldsymbol{u}-\boldsymbol{u}^*$.
2. Estimate local curvature *H* (log-synergism Hessian). Using controlled perturbations or local quadratic regression in log space (Eq. (14), obtain $(\boldsymbol{g},H)$ and check that residuals in *z=łog y* are small in the perturbation neighborhood.
3. Estimate input fluctuations *Σ* *in log space.* From samples $\{\boldsymbol{u}_t\}$ near $\boldsymbol{u}^*$, compute $\Sigma \approx \frac{1}{T-1}\sum _t \delta \boldsymbol{u}_t\delta \boldsymbol{u}_t^\top$; use diagonal/shrinkage estimates when *T* is limited.
4. Estimate observation noise $s^2$ *in log-output.* With replicates at fixed inputs, compute within-condition variance of $z^{(r)}=\log y^{(r)}$; alternatively use the residual variance from the quadratic fit.
5. Evaluate the bound and decide. Plug $(H,\Sigma ,s^2)$ into Eq. (10); choose a domain-relevant tolerance $\tau _{\textrm{KL}}$, and if the predicted risk (or its upper uncertainty bound) exceeds $\tau _{\textrm{KL}}$, include interaction terms (BST2) or redesign perturbations along high-risk directions.
6. Optional: attach an uncertainty bar for the predicted risk. Using covariance estimates for $(\hat{H},\hat{\Sigma },\hat{s}^2)$ (or a Cramér–Rao lower bound) and a delta-method propagation, report $\mathbb {E}[D_{\textrm{KL}}]\pm 1.96\,\textrm{SE}$; see Supplementary Sect. S5.2.

### Data-driven estimation of log-synergism (Hessian) *H*

Our main result, Eq. (10), requires (i) the local quadratic curvature *H* of the log-response $\mu (\boldsymbol{u})$ in log-coordinates and (ii) the input fluctuation covariance *Σ*. In practice, these quantities can be estimated from perturbation data around an operating point.

Let $\boldsymbol{u}=\log \boldsymbol{x}$ (componentwise) and fix an operating point $\boldsymbol{u}_0$. For sufficiently small perturbations $\delta \boldsymbol{u}=\boldsymbol{u}-\boldsymbol{u}_0$, fit the local quadratic model

14 $$\begin{aligned} \mu (\boldsymbol{u})\approx \mu _0+\boldsymbol{g}^\top \delta \boldsymbol{u}+\frac{1}{2}\delta \boldsymbol{u}^\top H\,\delta \boldsymbol{u}. \end{aligned}$$

There are two common estimation routes.

*Finite-difference estimation (controlled perturbations).* If one can measure $\mu (\boldsymbol{u})$ at designed perturbations (e.g. in silico experiments or controlled laboratory conditions), then central differences yield stable estimates:

15 $$\begin{aligned} g_i&\approx \frac{\mu (\boldsymbol{u}_0+\Delta \boldsymbol{e}_i)-\mu (\boldsymbol{u}_0-\Delta \boldsymbol{e}_i)}{2\Delta },\end{aligned}$$

16 $$\begin{aligned} H_{ii}&\approx \frac{\mu (\boldsymbol{u}_0+\Delta \boldsymbol{e}_i)-2\mu (\boldsymbol{u}_0)+\mu (\boldsymbol{u}_0-\Delta \boldsymbol{e}_i)}{\Delta ^2},\end{aligned}$$

17 $$\begin{aligned} H_{ij}&\approx \frac{1}{4\Delta ^2}\Big [\mu (\boldsymbol{u}_0+\Delta \boldsymbol{e}_i+\Delta \boldsymbol{e}_j)-\mu (\boldsymbol{u}_0+\Delta \boldsymbol{e}_i-\Delta \boldsymbol{e}_j)\end{aligned}$$

18 $$\begin{aligned}&\qquad \qquad -\mu (\boldsymbol{u}_0-\Delta \boldsymbol{e}_i+\Delta \boldsymbol{e}_j)+\mu (\boldsymbol{u}_0-\Delta \boldsymbol{e}_i-\Delta \boldsymbol{e}_j)\Big ]\quad (i\ne j), \end{aligned}$$

where $\boldsymbol{e}_i$ denotes the *i*-th coordinate vector and *Δ* is chosen within the local (approximately quadratic) regime.

*Regression estimation (noisy observational data).* Given *K* noisy measurements $\{(\delta \boldsymbol{u}^{(k)}, z^{(k)})\}_{k=1}^K$ with $z^{(k)}=\mu (\boldsymbol{u}_0+\delta \boldsymbol{u}^{(k)})+\eta ^{(k)}$, estimate $(\mu _0,\boldsymbol{g},H)$ via least squares (optionally ridge-regularized). A convenient parameterization is to stack all linear and (unique) quadratic terms into a vector $\boldsymbol{\phi }(\delta \boldsymbol{u})=(1,\delta u_1,\ldots ,\delta u_d, \delta u_1^2,\delta u_1\delta u_2,\ldots ,\delta u_d^2)$ and solve $\min _{\boldsymbol{\theta }}\sum _k (z^{(k)}-\boldsymbol{\theta }^\top \boldsymbol{\phi }(\delta \boldsymbol{u}^{(k)}))^2$. The estimated quadratic coefficients are then mapped back to a symmetric matrix *H* by construction. This regression route is particularly useful when perturbations are not axis-aligned or when replicates are available.

### Estimating input fluctuation statistics *Σ*

If input time series or replicate measurements $\boldsymbol{x}_t$ are available at the operating condition, work in log space $\boldsymbol{u}_t=\log \boldsymbol{x}_t$ and compute centered fluctuations $\delta \boldsymbol{u}_t=\boldsymbol{u}_t-\bar{\boldsymbol{u}}$. Then estimate the covariance of log-input fluctuations by

19 $$\begin{aligned} \Sigma \approx \frac{1}{T-1}\sum _{t=1}^{T} \delta \boldsymbol{u}_t\,\delta \boldsymbol{u}_t^\top . \end{aligned}$$

When *T* is small relative to the input dimension, a simple and robust option is a diagonal approximation (keeping only variances) or a shrinkage estimate $\Sigma _\alpha =(1-\alpha )\Sigma +\alpha \,\textrm{diag}(\Sigma )$ with $\alpha \in [0,1]$.

### Estimating the observation noise scale $s^2$

The closed form in Eq. (10) depends on the observational noise level $s^2$ in the Gaussian model, Eq. (6). When replicate measurements of the output are available at (approximately) fixed inputs, a simple estimate is the within-condition variance in *log*-output,

$$s^2 \approx \frac{1}{R-1}\sum _{r=1}^{R}\bigl (z^{(r)}-\bar{z}\bigr )^2, \qquad z^{(r)}=\log y^{(r)}.$$

Alternatively, when *H* and $\boldsymbol{g}$ are estimated by the local quadratic regression (Eq. 14), one may use the residual variance in *z* as an effective noise scale. If measurement noise is specified on the linear scale (additive or multiplicative noise in *y*), the induced noise in *z=łog y* is generally heteroscedastic; in that case, use a local noise model or bootstrap-based error propagation.

### Workflow for diagnosing the validity of BST1

Given *(H,Σ )* and the observation noise level *s* in Eq. (6), compute the expected KL risk using Eq. (10). Given a user-specified tolerance $\tau _{\textrm{KL}}$, declare BST1 sufficient when the predicted risk $\widehat{R}_{\textrm{KL}}$ is below $\tau _{\textrm{KL}}$, or conservatively when $\widehat{R}_{\textrm{KL}}+1.96\,\textrm{SE}<\tau _{\textrm{KL}}$. Conversely, a larger predicted risk flags regimes where mixed curvature (log-synergism) and/or strong fluctuations render BST1 unreliable, motivating second-order corrections or alternative reduced models. Importantly, even when *Σ* is diagonal (uncorrelated inputs), off-diagonal elements of *H* still contribute through $\textrm{tr}[(H\Sigma )^2]$ in Eq. (10), enabling detection of interaction-driven failure modes.

## Supplementary Information

Supplementary Information.

## Data Availability

All data generated or analysed during this study are included in this published article and its Supplementary Information files. Supplementary Information is available for this paper. It provides (i) a detailed step-by-step derivation and proof of the closed-form expected KL-risk criterion, (ii) an extension beyond Gaussian input fluctuations via a fourth-cumulant correction, and (iii) additional numerical robustness checks (Supplementary Figs. S1–S4).

## Code availability

The numerical and figure-generation scripts included in the submission package are sufficient to reproduce the figures used in this study.

## Declarations

### Use of AI tools

We used ChatGPT (OpenAI) to assist with drafting and editing parts of the manuscript text and for checking the clarity of explanations. All generated suggestions were reviewed by the author, and the final content, interpretations, and any errors remain the author’s responsibility.
