# Supplementary material for: Expected KL risk quantifies when first-order power-law approximations are sufficient
Source: Sci Rep. 2026 May 27;16:24302. doi: 10.1038/s41598-026-55101-y (PMC13444063; doi:10.1038/s41598-026-55101-y)
Supplement: Supplementary file 1 [file 41598_2026_55101_MOESM1_ESM.pdf]

Supplementary Information for: Expected KL risk quantifies when first-order power-law approximations are sufficient

Chikoo Oosawa

April 11, 2026

This Supplementary Information provides detailed derivations, extensions, and additional robustness checks supporting the main text.

Main message (in three bullets).

- **What is being quantified:** the leading-order *expected* predictive mismatch (KL risk) caused by using a first-order (multiplicative) BST power-law approximation.
- **What controls the mismatch:** only local *log-curvature* (Salvador's log-synergism Hessian H), input-fluctuation covariance Σ, and observation-noise variance s^2.
- **What you can do with it:** assess when "BST1" is adequate (or when "BST2"/interaction terms are needed) from local estimates of (H, Σ, s^2), within the local small-fluctuation regime.

S1 Introduction

Power-law representations and log-linearizations are ubiquitous in biochemical network analysis [1, 2, 3, 4, 5, 6]. For complementary reviews and practical perspectives on power-law/S-system modeling and related approximated kinetic representations, see Voit [7], Alves et al. [8, 9], and Sorribas and Savageau [10]. For a recent perspective on Savageau's foundational "Biochemical systems analysis" papers and subsequent developments in Biochemical Systems Theory, see Salvador [11]. In BST, a nonlinear rate law $v(\boldsymbol{x})$ is locally approximated near a reference state $\boldsymbol{x}^*$ by a multiplicative power-law form with exponents given by log-gains (elasticities). This approximation is convenient and often surprisingly accurate, but it discards the curvature of the log-response $\log v$ in log-coordinates. Salvador and Savageau introduced *log-synergism* to systematize the interpretation of this curvature via mixed second derivatives [12, 13].

Throughout, we work in log-coordinates $u = \log x$ and log-output $z = \log y$; steady-state (operating-point) analysis and small-signal gains are therefore taken in these log variables.

The present note asks a quantitative question: *What is the expected predictive cost of using a first-order (multiplicative) BST model when the true system exhibits log-synergism?* In statistical terms, model mismatch can be measured by the relative entropy (KL divergence) $D_{\mathrm{KL}}(p\|q)$ [14] between the true conditional distribution p and the approximate one q. We derive a closed-form leading-order expression for the *expected* KL risk, averaged over small input fluctuations, and show that it is fully determined by the local log-curvature $\boldsymbol{H}$ and the fluctuation covariance $\boldsymbol{\Sigma}$.

Contributions. Although the trace identity in Eq. (12) follows from classical Gaussian quadratic-form moments (Wick/Isserlis), this note contributes a BST-specific and practically usable risk criterion by: (a) casting the first-order BST approximation ("BST1") as a local flat (e-affine) projection in log-coordinates, where the curvature tensor is Salvador's log-synergism Hessian $\boldsymbol{H}$; (b) deriving a closed-form leading-order expression for the expected conditional KL risk under Gaussian input fluctuations with covariance $\boldsymbol{\Sigma}$ and homoscedastic Gaussian observation noise with variance s^2; (c) making the remainder order explicit under C^3 smoothness and small-fluctuation scaling; (d) recording a fourth-cumulant correction that extends the criterion to non-Gaussian input fluctuations; and (e) summarizing an operational workflow for estimating $(\boldsymbol{H}, \boldsymbol{\Sigma}, s^2)$ and using Eq. (S17) as a data-driven diagnostic for when interaction terms ("BST2") are warranted.

S2 BST first-order log–Taylor approximation

Let $u_j = \log x_j$ denote log-coordinates and write $\boldsymbol{u} = \log \boldsymbol{x}$ (componentwise). A first-order log–Taylor expansion of $\log v(\boldsymbol{u})$ around $\boldsymbol{u}^* = \log \boldsymbol{x}^*$ gives

$$\log v(\boldsymbol{u}) = \log v(\boldsymbol{u}^*) + \sum_{j=1}^{d} g_j \,(u_j - u_j^*) + \mathcal{O}(\|\boldsymbol{u} - \boldsymbol{u}^*\|^2), \tag{S1}$$

with *log-gains*

$$g_j = \left.\frac{\partial \log v}{\partial \log x_j}\right|_{\boldsymbol{x}^*} = \left.\frac{\partial \log v}{\partial u_j}\right|_{\boldsymbol{u}^*}. \tag{S2}$$

Exponentiating (S1) yields the BST power-law approximation

$$v(\boldsymbol{x}) \approx v(\boldsymbol{x}^*) \prod_{j=1}^{d} \left(\frac{x_j}{x_j^*}\right)^{g_j}. \tag{S3}$$

This is the local building block behind S-systems [1, 2, 3, 4, 5, 6].

S3 Log-synergism as curvature in log-coordinates

The curvature discarded by the first-order approximation is captured by the Hessian of the log-response, evaluated at the operating point:

$$H_{jk} := \left.\frac{\partial^2 \log v}{\partial u_j \,\partial u_k}\right|_{\boldsymbol{u}^*} = \left.\frac{\partial^2 \log v}{\partial \log x_j \,\partial \log x_k}\right|_{\boldsymbol{x}^*}. \tag{S4}$$

Mixed components H_{jk} ($j \neq k$) quantify non-multiplicative interactions in log-coordinates; these are the differential limit of Salvador's log-synergism [12, 13]. If $\log v(\boldsymbol{u})$ is exactly affine in $\boldsymbol{u}$, then $\boldsymbol{H} = \boldsymbol{0}$ and (S3) is locally exact.

From an information-geometric viewpoint [15, 16, 17, 18], the first-order approximation (S3) can be regarded as a local *flat (e-affine) projection* of the log-response onto its tangent hyperplane at $\boldsymbol{u}^*$. Retaining the quadratic term in (S5) yields a minimal *curvature correction* (sometimes called a second-order BST approximation), so the gap between "BST1" and "BST2" is precisely the log-curvature tensor $\boldsymbol{H}$. The main theorem below quantifies the information-theoretic cost of discarding this curvature under small input fluctuations.

S4 KL risk for a first-order approximation: setup

To connect with the preceding rate-law notation, you may regard the scalar response $y(\boldsymbol{u})$ as the quantity of interest (e.g., $y(\boldsymbol{u}) = v(\boldsymbol{x})$ evaluated at $\boldsymbol{x} = \exp(\boldsymbol{u})$), so that $\mu(\boldsymbol{u}) = \log y(\boldsymbol{u})$. Let the *log-output* be modeled by

$$\mu_{\mathrm{BST2}}(\boldsymbol{u}) := \log y(\boldsymbol{u}) = a + \boldsymbol{g}^{\mathsf{T}}\delta\boldsymbol{u} + \frac{1}{2}\,\delta\boldsymbol{u}^{\mathsf{T}}\boldsymbol{H}\,\delta\boldsymbol{u} + \mathcal{O}(\|\delta\boldsymbol{u}\|^3), \qquad \delta\boldsymbol{u} := \boldsymbol{u} - \boldsymbol{u}^*, \tag{S5}$$

where $\boldsymbol{g} = \nabla_{\boldsymbol{u}}\mu_{\mathrm{BST2}}(\boldsymbol{u}^*)$ and $\boldsymbol{H} = \nabla^2_{\boldsymbol{u}}\mu_{\mathrm{BST2}}(\boldsymbol{u}^*)$. The first-order BST model drops the quadratic term:

$$\mu_{\mathrm{BST1}}(\boldsymbol{u}) = a + \boldsymbol{g}^{\mathsf{T}}\delta\boldsymbol{u}. \tag{S6}$$

S4.1 Optional context: information thermodynamics and time irreversibility

The main text interprets KL divergence as an *excess predictive loss* between conditional output models at a fixed operating point. For completeness, we note that in trajectory-level nonequilibrium settings, KL divergences between forward and time-reversed *path* measures quantify irreversibility and dissipated work under standard assumptions, for example

$$\langle W_{\mathrm{diss}}\rangle = k_B T\, D_{\mathrm{KL}}(P_F \| P_R), \tag{S7}$$

see, e.g., [42, 43]. More generally, for interacting subsystems with explicit causal structure, generalized fluctuation theorems and second-law inequalities incorporate information-flow terms on Bayesian (causal) networks [49], and information-geometric formulations of stochastic interactions and entropy production [50]. We do *not* claim that Eq. (S7) applies directly to the static, conditional KL risk used in the main text; connecting the two requires an explicit stochastic dynamical model and a clearly defined coarse-graining map from microscopic degrees of freedom to observables. Nevertheless, the present KL-risk criterion can be viewed as a conservative, data-driven diagnostic of information loss due to neglected interactions, and it could be combined with trajectory-based "arrow-of-time" estimators in future work. Indeed, several experimental and biophysical studies estimate irreversibility/entropy production from coarse-grained time series (including tracking-based observables), while emphasizing that coarse-graining typically yields lower bounds [44, 45, 46, 47, 48].

S4.2 Observation model and conditional KL divergence

Assume the observed log-output $z = \log y$ is corrupted by homoscedastic Gaussian noise with variance s^2. This additive Gaussian-noise model in log space is a standard closure that yields an explicit conditional KL. If measurement noise is specified on the linear scale (for example, additive or multiplicative noise in y), the induced noise in $z = \log y$ is generally heteroscedastic and may be non-Gaussian; in that case, one may replace Eq. (S9) by the appropriate conditional KL or use a local Fisher-information approximation. We emphasize that both models share the same noise variance s^2; this isolates the contribution of mean mismatch (if variances differ, additional variance-ratio and log terms enter the KL divergence).

$$p(z \mid \boldsymbol{u}) = \mathcal{N}(z; \mu_{\mathrm{BST2}}(\boldsymbol{u}), s^2), \qquad q(z \mid \boldsymbol{u}) = \mathcal{N}(z; \mu_{\mathrm{BST1}}(\boldsymbol{u}), s^2). \tag{S8}$$

For two univariate Gaussians with the *same* variance s^2, the KL divergence is explicit:

$$D_{\mathrm{KL}}(p(\cdot \mid \boldsymbol{u}) \,\|\, q(\cdot \mid \boldsymbol{u})) = \frac{\big(\mu_{\mathrm{BST2}}(\boldsymbol{u}) - \mu_{\mathrm{BST1}}(\boldsymbol{u})\big)^2}{2s^2}. \tag{S9}$$

(We include a short derivation in Appendix A.)

Remark (Vector-valued outputs). The setup above is written for a scalar log-output $z = \log y$ for clarity. If instead the observation is vector-valued $z \in \mathbb{R}^m$ and both models share the same Gaussian noise covariance $\boldsymbol{S}$ (i.e., $p(z \mid \boldsymbol{u}) = \mathcal{N}(z; \boldsymbol{m}_2(\boldsymbol{u}), \boldsymbol{S})$ and $q(z \mid \boldsymbol{u}) = \mathcal{N}(z; \boldsymbol{m}_1(\boldsymbol{u}), \boldsymbol{S}))$, then the conditional KL divergence is

$$D_{\mathrm{KL}}(p\|q) = \frac{1}{2}\,(\boldsymbol{m}_2 - \boldsymbol{m}_1)^{\mathsf{T}} \boldsymbol{S}^{-1} (\boldsymbol{m}_2 - \boldsymbol{m}_1).$$

For isotropic noise $\boldsymbol{S} = s^2 \boldsymbol{I}$ (and independent noise components), the KL reduces to a sum of scalar equal-variance terms, so the same trace contractions in Theorem 1 apply componentwise.

Remark (Fisher-information interpretation). This remark links our KL criterion to the Fisher–Rao metric; the explicit identities below use the homoscedastic Gaussian observation model (S8). More generally, for a regular parametric family $p(z \mid \theta)$, the KL divergence admits the local quadratic expansion

$$D_{\mathrm{KL}}(p(\cdot \mid \theta) \,\|\, p(\cdot \mid \theta + \delta\theta)) = \frac{1}{2}\,\delta\theta^{\mathsf{T}} \boldsymbol{I}(\theta)\,\delta\theta + o(\|\delta\theta\|^2), \tag{S10}$$

where $\boldsymbol{I}(\theta)$ is the Fisher information matrix, which defines the canonical Riemannian metric (the Fisher–Rao metric) in information geometry [15, 16, 17, 18, 19, 20, 25, 26, 27]. This connection is also central to recent Fisher-information-based speed-limit inequalities and hidden-state diagnostics in stochastic dynamics [51], and to information-geometric inequalities in chemical thermodynamics [52]. In our homoscedastic Gaussian observation model (S8), taking $\theta = \mu$ (mean parameter) gives $\boldsymbol{I}(\mu) = 1/s^2$, and the identity (S9) is exact:

$$D_{\mathrm{KL}}(\mathcal{N}(\mu, s^2) \,\|\, \mathcal{N}(\nu, s^2)) = \frac{1}{2}\,\boldsymbol{I}(\mu)\,(\mu - \nu)^2 = \frac{(\mu - \nu)^2}{2s^2}. \tag{S11}$$

Thus, the expected KL risk in Theorem 1 can be interpreted as a Fisher–Rao (mean-parameter) metric applied to the second-order mean mismatch $\Delta\mu(\boldsymbol{u})$ induced by discarding the log-curvature $\boldsymbol{H}$.

Remark (Wasserstein interpretation). This remark is specific to the same homoscedastic Gaussian observation model (S8); for unequal variances/covariances, D_{KL} and W_2^2 differ and include distinct covariance terms. For the homoscedastic Gaussian observation model (S8), the conditional KL divergence (S9) is proportional to the squared 2-Wasserstein distance. Indeed, for Gaussians with identical variance s^2,

$$W_2^2(\mathcal{N}(\mu, s^2), \mathcal{N}(\nu, s^2)) = (\mu - \nu)^2, \tag{S12}$$

a special case of the closed-form formula for W_2 between Gaussian measures [28, 29] (see also [30, 31, 32]). Hence (S9) can be rewritten as

$$D_{\mathrm{KL}}(p(\cdot \mid \boldsymbol{u}) \,\|\, q(\cdot \mid \boldsymbol{u})) = \frac{1}{2s^2}\, W_2^2(p(\cdot \mid \boldsymbol{u}), q(\cdot \mid \boldsymbol{u})), \tag{S13}$$

so our expected KL risk can be read as a noise-scaled expected squared Wasserstein mismatch in this Gaussian setting. For unequal variances/covariances, D_{KL} and W_2^2 generally differ and contain distinct covariance terms [29].

Define the local mismatch

$$\Delta\mu(\boldsymbol{u}) := \mu_{\mathrm{BST2}}(\boldsymbol{u}) - \mu_{\mathrm{BST1}}(\boldsymbol{u}) = \frac{1}{2}\,\delta\boldsymbol{u}^{\mathsf{T}} \boldsymbol{H}\,\delta\boldsymbol{u} + \mathcal{O}(\|\delta\boldsymbol{u}\|^3). \tag{S14}$$

Then (S9) yields, to leading order,

$$D_{\mathrm{KL}}(p(\cdot \mid \boldsymbol{u}) \,\|\, q(\cdot \mid \boldsymbol{u})) = \frac{1}{2s^2}\left(\frac{1}{2}\,\delta\boldsymbol{u}^{\mathsf{T}} \boldsymbol{H}\,\delta\boldsymbol{u}\right)^2 + \mathcal{O}(\|\delta\boldsymbol{u}\|^5). \tag{S15}$$

S4.3 Input fluctuations

Assume small log-input fluctuations around the operating point:

$$\delta \boldsymbol{u} \sim \mathcal{N}(\mathbf{0}, \boldsymbol{\Sigma}), \tag{S16}$$

with covariance $\boldsymbol{\Sigma} = \mathbb{E}[\delta \boldsymbol{u}\, \delta \boldsymbol{u}^\mathsf{T}]$.

S5 Main result: expected KL risk controlled by log-synergism

We now compute the *expected* KL risk $\mathbb{E}_{\delta \boldsymbol{u}}[D_{\mathrm{KL}}(p\|q)]$ under (S16).

S5.1 Theorem (closed-form leading order)

Assumption (Taylor remainder and scaling). Assume $\mu_{\mathrm{BST2}}(\boldsymbol{u})$ is C^3 in a neighborhood of $\boldsymbol{u}^*$, so that the remainder in (S5) obeys a local bound $|R_3(\delta \boldsymbol{u})| \le C_3 \|\delta \boldsymbol{u}\|^3$ for $\|\delta \boldsymbol{u}\| \le \rho$. Under the small-fluctuation scaling $\delta \boldsymbol{u} = \boldsymbol{\Sigma}^{1/2} \xi$ with $\xi = \mathcal{O}(1)$, moments satisfy $\mathbb{E}[\|\delta \boldsymbol{u}\|^k] = \mathcal{O}(\|\boldsymbol{\Sigma}\|^{k/2})$ (e.g., for Gaussian inputs). Here $\|\cdot\|$ denotes the spectral norm; equivalently, one may parameterize $\boldsymbol{\Sigma} = \varepsilon^2 \boldsymbol{\Sigma}_0$ with $\varepsilon \ll 1$ and $\boldsymbol{\Sigma}_0 = \mathcal{O}(1)$.

Remark (Gaussian tails and local validity). The bound $|R_3(\delta \boldsymbol{u})| \le C_3 \|\delta \boldsymbol{u}\|^3$ is assumed to hold locally for $\|\delta \boldsymbol{u}\| \le \rho$, whereas the Gaussian input model has unbounded support. Under the small-fluctuation scaling $\boldsymbol{\Sigma} = \varepsilon^2 \boldsymbol{\Sigma}_0$ with $\varepsilon \ll 1$, the tail probability $\mathbb{P}(\|\delta \boldsymbol{u}\| > \rho) = \mathbb{P}(\|\xi\| > \rho/\varepsilon)$ decays rapidly as $\varepsilon \to 0$, so the contribution of the tail region to $\mathbb{E}[D_{\mathrm{KL}}]$ is negligible compared with the leading term in Eq. (S17). Equivalently, one may interpret the theorem as applying to truncated (local) input fluctuations within the neighborhood where the log–Taylor expansion is valid.

Theorem 1. *Let $\delta \boldsymbol{u} \sim \mathcal{N}(\mathbf{0}, \boldsymbol{\Sigma})$ and assume the Gaussian observation model* (S8)*. Then the expected conditional KL divergence satisfies*

$$\mathbb{E}_{\delta \boldsymbol{u}}\left[D_{\mathrm{KL}}(p(\cdot \mid \boldsymbol{u}) \,\|\, q(\cdot \mid \boldsymbol{u}))\right] = \frac{1}{8s^2} \left\{ 2\,\mathrm{tr}[(\boldsymbol{H}\boldsymbol{\Sigma})^2] + \left(\mathrm{tr}(\boldsymbol{H}\boldsymbol{\Sigma})\right)^2 \right\} + \mathcal{O}\left(\|\boldsymbol{\Sigma}\|^{5/2}\right). \tag{S17}$$

Remark (connection to classical Gaussian quadratic-form moments). The trace combination in (S17) comes from the standard fourth-moment identity for Gaussian quadratic forms (Wick/Isserlis), widely used in multivariate statistics and random matrix calculations; see, e.g., [33, 34, 35]. The present note emphasizes its interpretation as a *leading-order expected KL risk* for the first-order BST (multiplicative power-law) approximation, with the curvature tensor $\boldsymbol{H}$ identified with Salvador's log-synergism Hessian.

S5.2 Proof (step-by-step)

Starting from (S9) and (S14), the leading-order term is

$$D_{\mathrm{KL}}(p\|q) = \frac{1}{2s^2} \left(\frac{1}{2}\, \delta \boldsymbol{u}^\mathsf{T} \boldsymbol{H}\, \delta \boldsymbol{u} \right)^2 + \text{higher order}.$$

Thus

$$\mathbb{E}[D_{\mathrm{KL}}(p\|q)] = \frac{1}{8s^2}\, \mathbb{E}\left[\left(\delta \boldsymbol{u}^\mathsf{T} \boldsymbol{H}\, \delta \boldsymbol{u} \right)^2 \right] + \mathcal{O}\left(\|\boldsymbol{\Sigma}\|^{5/2}\right), \tag{S18}$$

where the remainder reflects that the cubic term in (S14) contributes at order $\mathbb{E}[\|\delta \boldsymbol{u}\|^5] \sim \mathcal{O}(\|\boldsymbol{\Sigma}\|^{5/2})$ and the cross term between quadratic and cubic contributes $\mathcal{O}(\|\boldsymbol{\Sigma}\|^{5/2})$ under small-fluctuation scaling.

It remains to compute $\mathbb{E}[(\delta\boldsymbol{u}^\top \boldsymbol{H}\,\delta\boldsymbol{u})^2]$. Write $Q := \delta\boldsymbol{u}^\top \boldsymbol{H}\,\delta\boldsymbol{u} = \sum_{i,j} H_{ij}\,\delta u_i\,\delta u_j$. Then

$$\begin{aligned}\mathbb{E}[Q^2] &= \mathbb{E}\left[\sum_{i,j}\sum_{k,\ell} H_{ij}H_{k\ell}\,\delta u_i \delta u_j \delta u_k \delta u_\ell\right] \\ &= \sum_{i,j}\sum_{k,\ell} H_{ij}H_{k\ell}\,\mathbb{E}[\delta u_i \delta u_j \delta u_k \delta u_\ell]. \end{aligned} \tag{S19}$$

For a zero-mean multivariate Gaussian, Wick/Isserlis gives (Appendix B)

$$\mathbb{E}[\delta u_i \delta u_j \delta u_k \delta u_\ell] = \Sigma_{ij}\Sigma_{k\ell} + \Sigma_{ik}\Sigma_{j\ell} + \Sigma_{i\ell}\Sigma_{jk}.$$

Substituting into (S19) and rearranging indices yields

$$\mathbb{E}[Q^2] = 2\,\mathrm{tr}[(\boldsymbol{H\Sigma})^2] + \big(\mathrm{tr}(\boldsymbol{H\Sigma})\big)^2. \tag{S20}$$

Combining (S18) with (S20) proves (S17). □

S5.3 Interpretation

Equation (S17) shows that the predicted loss couples (i) the *interaction curvature* $\boldsymbol{H}$ and (ii) the *fluctuation statistics* $\boldsymbol{\Sigma}$. Even when inputs are uncorrelated (diagonal $\boldsymbol{\Sigma}$), mixed log-curvature entries H_{jk} contribute through $\mathrm{tr}[(\boldsymbol{H\Sigma})^2]$.
In other words, under the local quadratic mismatch $\Delta\mu(\boldsymbol{u}) \approx \frac{1}{2}\,\delta\boldsymbol{u}^\top \boldsymbol{H}\,\delta\boldsymbol{u}$, the expected risk reduces to a moment of a quadratic form, which is why the same trace structure appears broadly beyond BST whenever Gaussian fluctuations are paired with a second-order model mismatch (see the remark after Theorem 1).

For non-Gaussian input fluctuations, Appendix C provides the leading fourth-cumulant correction (Eqs. (S22)–(S24)), so the criterion can be adjusted using an empirical estimate of the kurtosis tensor κ when Gaussianity is not a good approximation.

S6 Practical use, robustness, and common extensions

S6.1 Operational workflow from data

Equation (S17) is intended as an *operational* diagnostic: it translates local log-curvature (log-synergism) and measured fluctuation statistics into an expected predictive mismatch. A minimal workflow is:

1. Choose an operating point $\boldsymbol{u}^*$ (e.g., a steady state, a reference condition, or the mean of a local experimental condition) and form centered log-inputs $\delta\boldsymbol{u} = \boldsymbol{u} - \boldsymbol{u}^*$.

2. Estimate the local quadratic expansion of $\mu(\boldsymbol{u}) = \log y(\boldsymbol{u})$ near $\boldsymbol{u}^*$ via a (possibly weighted) least-squares fit $\mu(\boldsymbol{u}) \approx a + \boldsymbol{g}^\top\delta\boldsymbol{u} + \frac{1}{2}\,\delta\boldsymbol{u}^\top \boldsymbol{H}\,\delta\boldsymbol{u}$, then symmetrize $\boldsymbol{H} \leftarrow (\boldsymbol{H} + \boldsymbol{H}^\top)/2$.

3. Estimate the log-input covariance $\boldsymbol{\Sigma} = \mathbb{E}[\delta\boldsymbol{u}\,\delta\boldsymbol{u}^\top]$ from replicate measurements or from a stationary time window around $\boldsymbol{u}^*$.

4. Estimate the observation-noise variance s^2 in log space (e.g., from technical replicates or from the residual variance of the local fit when input variation is controlled).

5. Compute the predicted risk $\mathbb{E}[D_{\mathrm{KL}}]$ from Eq. (S17) and compare it to an application-specific tolerance (e.g., a "small" expected mismatch per condition).

S6.2 Error bars for the estimated $\mathbb{E}[D_{\mathrm{KL}}]$ (delta method and Cramér–Rao lower bound)

In applications, $(\boldsymbol{H}, \boldsymbol{\Sigma}, s^2)$ are estimated from finite data, so the predicted risk

$$\widehat{\mathbb{E}[D_{\mathrm{KL}}]} := \mathcal{R}(\hat{\boldsymbol{H}}, \hat{\boldsymbol{\Sigma}}, \hat{s}^2), \qquad \mathcal{R}(\boldsymbol{H}, \boldsymbol{\Sigma}, s^2) = \frac{1}{8s^2}\Big\{2\mathrm{tr}[(\boldsymbol{H}\boldsymbol{\Sigma})^2] + \big(\mathrm{tr}(\boldsymbol{H}\boldsymbol{\Sigma})\big)^2\Big\},$$

should ideally be reported with an uncertainty bar. A convenient large-sample approximation is the delta method [23]:

$$\mathrm{Var}(\widehat{\mathbb{E}[D_{\mathrm{KL}}]}) \approx \nabla_\theta \mathcal{R}(\theta)^\top \, \mathrm{Cov}(\hat{\theta}) \, \nabla_\theta \mathcal{R}(\theta), \tag{S21}$$

where θ stacks the unique entries of $(\boldsymbol{H}, \boldsymbol{\Sigma})$ and s^2; one may evaluate (S21) at plug-in estimates. An analytic differential form is

$$d\mathcal{R} = \frac{1}{8s^2}\mathrm{tr}\big(\boldsymbol{G}^\top (d\boldsymbol{H}\,\boldsymbol{\Sigma} + \boldsymbol{H}\,d\boldsymbol{\Sigma})\big) - \frac{\mathcal{R}}{s^2}\,ds^2, \qquad \boldsymbol{G} := 4(\boldsymbol{H}\boldsymbol{\Sigma})^\top + 2\mathrm{tr}(\boldsymbol{H}\boldsymbol{\Sigma})\boldsymbol{I},$$

so gradients with respect to $\boldsymbol{H}$, $\boldsymbol{\Sigma}$, and s^2 are obtained immediately (or computed numerically if preferred).

Covariance inputs and a CRB-based lower bound. For the local quadratic regression $z = \Phi\theta + \varepsilon$ with $\varepsilon \sim \mathcal{N}(0, s^2)$, the Cramér–Rao bound gives $\mathrm{Cov}(\hat{\theta}) \succeq s^2(\Phi^\top\Phi)^{-1}$, hence a lower bound on the achievable variance contribution from estimating $\boldsymbol{H}$ [20, 21, 22]. For $\hat{\boldsymbol{\Sigma}}$ from T log-input samples, under a Gaussian/Wishart approximation,

$$\mathrm{Cov}(\hat{\Sigma}_{ij}, \hat{\Sigma}_{kl}) \approx \frac{1}{T-1}\Big(\Sigma_{ik}\Sigma_{jl} + \Sigma_{il}\Sigma_{jk}\Big),$$

and for $\hat{s}^2$ from R replicate log-output measurements, $\mathrm{Var}(\hat{s}^2) \approx 2s^4/(R-1)$. Combining these pieces in (S21) yields an approximate standard error and a corresponding error bar (e.g., $\widehat{\mathbb{E}[D_{\mathrm{KL}}]} \pm 1.96\,\mathrm{SE}$). When sample sizes are small or assumptions are questionable, bootstrap-based error propagation provides a robust alternative.

Local validity check. Because the derivation is local, a practical sanity check is that typical fluctuations are small relative to the radius of validity of the log–Taylor expansion. For example, one may require $\sqrt{\mathrm{tr}(\boldsymbol{\Sigma})} \ll \rho$ (or directly estimate a tail probability $\Pr(\|\delta\boldsymbol{u}\| > \rho)$ in a truncated analysis) so that the neighborhood where the quadratic approximation is accurate dominates the expectation.

S6.3 Minimal numerical check (toy quadratic model)

To illustrate Eq. (S17), consider a purely quadratic log-response (no remainder) with $d = 3$ and

$$\boldsymbol{H} = \begin{pmatrix} 0 & 0.7 & -0.2 \\ 0.7 & 0.3 & 0.5 \\ -0.2 & 0.5 & -0.1 \end{pmatrix}, \qquad \boldsymbol{\Sigma} = \varepsilon^2 \begin{pmatrix} 1 & 0.4 & 0.2 \\ 0.4 & 1 & 0.3 \\ 0.2 & 0.3 & 1 \end{pmatrix}, \qquad \varepsilon = 0.15, \qquad s = 0.1.$$

For this model, $\Delta\mu(\boldsymbol{u}) = \frac{1}{2}\,\delta\boldsymbol{u}^\top \boldsymbol{H}\,\delta\boldsymbol{u}$ and $D_{\mathrm{KL}}(p\|q) = (\delta\boldsymbol{u}^\top\boldsymbol{H}\,\delta\boldsymbol{u})^2/(8s^2)$ holds exactly. Equation (S17) predicts

$$\mathbb{E}[D_{\mathrm{KL}}] = 0.0356577.$$

A Monte Carlo estimate with 4×10^5 samples from $\delta\boldsymbol{u} \sim \mathcal{N}(\boldsymbol{0}, \boldsymbol{\Sigma})$ gives $\mathbb{E}[D_{\mathrm{KL}}] \approx 0.035645 \pm 0.00019$ (standard error), consistent with the closed form.

S6.4 Limitations and reviewer-facing clarifications

The main result is deliberately minimal, and several assumptions are chosen to keep the criterion closed-form. Common points of clarification are:

- **Gaussian, homoscedastic observation noise.** Equation (S9) is exact only when both models share the same noise variance s^2 in log space. If noise is heteroscedastic or non-Gaussian (e.g., noise specified on the linear scale before taking logs), one may replace Eq. (S9) by the appropriate conditional KL or use the local Fisher-information approximation in Eq. (S10).
- **Small-fluctuation (local) regime.** Theorem 1 is a local statement. When fluctuations are not small, higher-order terms in $\Delta\mu$ and tail regions can contribute, and BST1 adequacy should be checked by direct simulation or by fitting beyond second order.
- **Input non-Gaussianity.** Appendix C records the leading fourth-cumulant correction. Empirically estimating the kurtosis tensor κ allows one to adjust the criterion when Gaussian inputs are not a good approximation.
- **Scalar output for exposition.** The scalar presentation emphasizes the role of $\boldsymbol{H}$. For vector outputs with shared covariance, the KL reduces to a quadratic form in the mean mismatch, and the same trace contractions apply componentwise for isotropic noise (see the remark in Section 4.1).
- **Wasserstein interpretation is conditional.** The proportionality between D_{KL} and W_2^2 in Eqs. (S12)–(S13) is specific to the equal-variance Gaussian setting and should be read as an interpretation rather than a general identity.

A KL divergence between equal-variance Gaussians

Let $p = \mathcal{N}(z; \mu, s^2)$ and $q = \mathcal{N}(z; \nu, s^2)$. Then

$$D_{\mathrm{KL}}(p\|q) = \int p(z) \log \frac{p(z)}{q(z)} \, \mathrm{d}z = \int p(z) \left[-\frac{(z-\mu)^2}{2s^2} + \frac{(z-\nu)^2}{2s^2} \right] \mathrm{d}z.$$

Expanding $(z-\nu)^2 = (z-\mu+\mu-\nu)^2 = (z-\mu)^2 + 2(z-\mu)(\mu-\nu) + (\mu-\nu)^2$ and using $\mathbb{E}_p[z-\mu] = 0$ gives

$$D_{\mathrm{KL}}(p\|q) = \frac{(\mu-\nu)^2}{2s^2},$$

which is (S9).

B Gaussian fourth-moment identity (Wick/Isserlis)

Let $\delta\boldsymbol{u} \sim \mathcal{N}(\mathbf{0}, \boldsymbol{\Sigma})$. Wick's theorem (Isserlis' formula) states that for zero-mean Gaussian variables,

$$\mathbb{E}[\delta u_i \delta u_j \delta u_k \delta u_\ell] = \Sigma_{ij}\Sigma_{k\ell} + \Sigma_{ik}\Sigma_{j\ell} + \Sigma_{i\ell}\Sigma_{jk}.$$

To connect this to traces, note that

$$\mathrm{tr}(\boldsymbol{H\Sigma}) = \sum_{i,j} H_{ij}\Sigma_{ji} = \sum_{i,j} H_{ij}\Sigma_{ij},$$

and

$$\mathrm{tr}[(\boldsymbol{H\Sigma})^2] = \sum_{i,j,k,\ell} H_{ij}\Sigma_{jk}H_{k\ell}\Sigma_{\ell i}.$$

Substituting Wick's identity into (S19) yields

$$\begin{aligned}\mathbb{E}[Q^2] &= \sum_{i,j,k,\ell} H_{ij}H_{k\ell}\Sigma_{ij}\Sigma_{k\ell} + \sum_{i,j,k,\ell} H_{ij}H_{k\ell}\Sigma_{ik}\Sigma_{j\ell} + \sum_{i,j,k,\ell} H_{ij}H_{k\ell}\Sigma_{i\ell}\Sigma_{jk} \\ &= \big(\mathrm{tr}(\boldsymbol{H\Sigma})\big)^2 + \mathrm{tr}\big[(\boldsymbol{H\Sigma})^2\big] + \mathrm{tr}\big[(\boldsymbol{H\Sigma})^2\big],\end{aligned}$$

where the last two terms are equal after index relabeling, giving (S20).

C Beyond Gaussian inputs: a fourth-cumulant correction

The leading-order risk (S18) depends on the fourth-order moment of $\delta\boldsymbol{u}$. If the input fluctuations are *non-Gaussian* but still centered and sufficiently small, the same reduction holds with $\mathbb{E}[\delta u_i \delta u_j \delta u_k \delta u_\ell]$ replaced by the true fourth moment. It is often convenient to separate the Gaussian part from the genuine non-Gaussianity via the *fourth cumulant* (tensor kurtosis)

$$\kappa_{ijkl} := \mathrm{cum}(\delta u_i, \delta u_j, \delta u_k, \delta u_\ell) = \mathbb{E}[\delta u_i \delta u_j \delta u_k \delta u_\ell] - \Big(\Sigma_{ij}\Sigma_{k\ell} + \Sigma_{ik}\Sigma_{j\ell} + \Sigma_{i\ell}\Sigma_{jk}\Big). \tag{S22}$$

Then the quadratic form $Q = \delta\boldsymbol{u}^\top \boldsymbol{H}\,\delta\boldsymbol{u}$ satisfies

$$\mathbb{E}[Q^2] = 2\,\mathrm{tr}\big[(\boldsymbol{H\Sigma})^2\big] + \big(\mathrm{tr}(\boldsymbol{H\Sigma})\big)^2 + \sum_{i,j,k,\ell} H_{ij}H_{k\ell}\,\kappa_{ijkl}. \tag{S23}$$

Consequently, the leading-order expected KL risk becomes

$$\mathbb{E}[D_{\mathrm{KL}}(p\|q)] = \frac{1}{8s^2}\Big\{2\,\mathrm{tr}\big[(\boldsymbol{H\Sigma})^2\big] + \big(\mathrm{tr}(\boldsymbol{H\Sigma})\big)^2 + \langle \boldsymbol{H}\otimes\boldsymbol{H}, \boldsymbol{\kappa}\rangle\Big\} + (\text{higher order}), \tag{S24}$$

where $\langle \boldsymbol{H}\otimes\boldsymbol{H}, \boldsymbol{\kappa}\rangle := \sum_{i,j,k,\ell} H_{ij}H_{k\ell}\kappa_{ijkl}$ denotes the double contraction. For Gaussian inputs, $\boldsymbol{\kappa} = \boldsymbol{0}$ and (S24) reduces to Theorem 1.

In high dimensions, the non-Gaussian term involves a contraction of the Hessian $\boldsymbol{H}$ with the kurtosis tensor $\boldsymbol{\kappa}$, which naively scales as $O(d^4)$ in both storage and computation. If $\boldsymbol{\kappa}$ (and/or $\boldsymbol{\Sigma}$) is approximately low-rank or admits a compact tensor representation, the same quantity can be computed via sequential contractions on the factorized form, potentially reducing the cost substantially without explicitly forming all $O(d^4)$ cumulant components [36]. We leave a systematic exploration of such tensor-structured accelerations for future work.

D Additional numerical robustness checks

To clarify the scope of the Gaussian-noise/Gaussian-fluctuation assumptions behind Eq. (S17), we include four additional numerical checks. These tests illustrate (i) sensitivity to heavy-tailed input fluctuations, (ii) robustness under general (correlated) covariance structure, (iii) the expected $1/s^2$ scaling with observation-noise level, and (iv) how mechanistic choices (e.g., inhibition type in an enzyme-rate example) can change mixed log-curvature and thus the predicted risk.

E Relation to metabolic control analysis

MCA characterizes the linear response of steady-state fluxes and concentrations to parameter perturbations [37, 38, 39, 40, 41]. Although BST and MCA share local log-derivatives (elasticities), the curvature discarded by a *first-order* approximation differs: BST1 discards the curvature of local rate laws (H), whereas a first-order MCA treatment discards the curvature of the steady-state input–output map. Clarifying this distinction is useful when interpreting information-thermodynamic costs of model reduction.

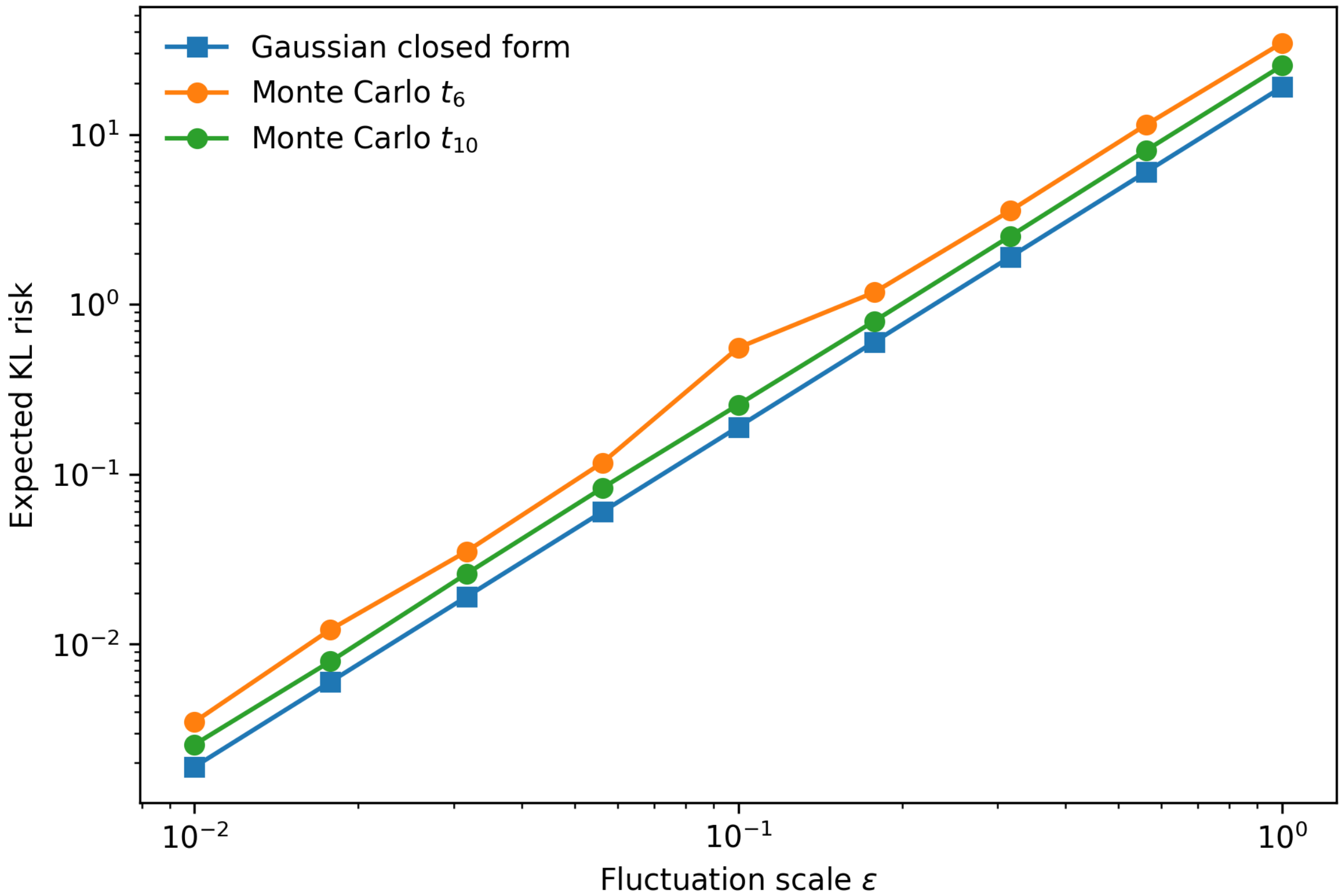

Figure S1: Heavy-tailed log-input fluctuations with matched covariance. We compare the Gaussian closed form (Eq. (S17)) to Monte Carlo estimates when $\delta\boldsymbol{u}$ follows a multivariate t distribution with degrees of freedom $\nu = 6$ and 10 (finite fourth moment). The scaling with the fluctuation amplitude ϵ remains consistent, while heavy tails increase the overall risk.

Relation to SKM and MRA. Beyond MCA, other local steady-state frameworks include structural kinetic modeling (SKM) and modular response analysis (MRA). SKM parameterizes the local Jacobian to probe dynamical capabilities and stability without committing to detailed rate laws, whereas MRA exploits modularity to infer local response coefficients from perturbation data. These approaches are complementary to our KL-risk criterion: rather than estimating elasticities per se, we quantify the information-theoretic cost of truncating nonlinear kinetics at first order in log-coordinates and use it for risk-controlled model reduction and perturbation design [53, 54].

References

[1] Savageau, M. A. Biochemical systems analysis. I. Some mathematical properties of the rate law for the component enzymatic reactions. *Journal of Theoretical Biology* **25**, 365–369 (1969). `https://doi.org/10.1016/S0022-5193(69)80026-3`

[2] Savageau, M. A. Biochemical systems analysis. II. The steady-state solutions for an n-pool system using a power-law approximation. *Journal of Theoretical Biology* **25**, 370–379 (1969). doi:10.1016/S0022-5193(69)80027-5.

[3] Savageau, M. A. Biochemical systems analysis. III. Dynamic solutions using a power-law approximation. *Journal of Theoretical Biology* **26**, 215–226 (1970). doi:10.1016/S0022-5193(70)80013-3.

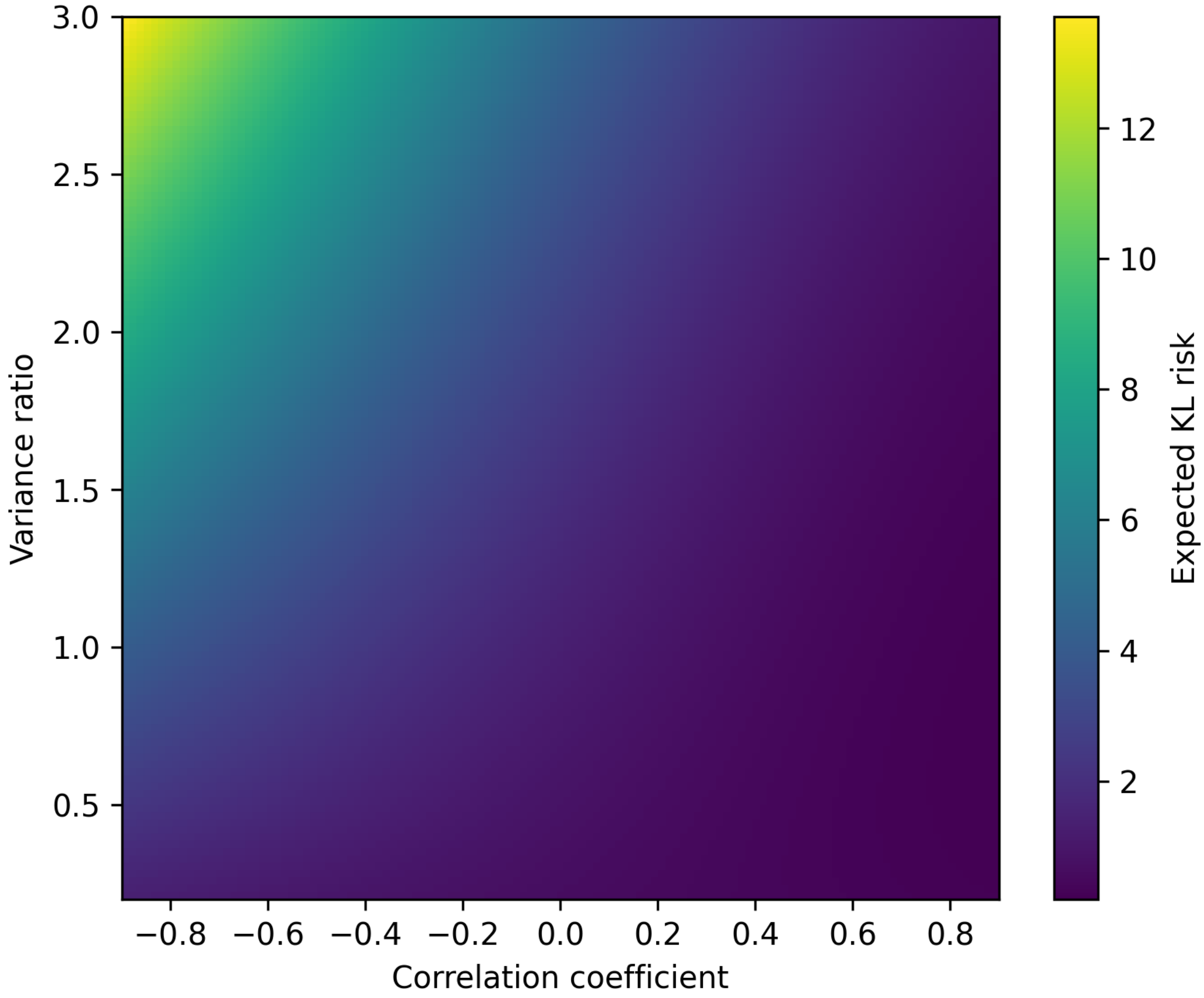

Figure S2: General (anisotropic/correlated) Gaussian covariances. Monte Carlo estimates match the closed form for several representative covariance structures, confirming that Eq. (S17) applies to general Σ (not only isotropic fluctuations).

[4] Savageau, M. A. *Biochemical Systems Analysis: A Study of Function and Design in Molecular Biology.* Addison-Wesley, Reading, MA (1976).

[5] Voit, E. O. *Computational Analysis of Biochemical Systems: A Practical Guide for Biochemists and Molecular Biologists.* Cambridge University Press (2000).

[6] Voit, E. O. Biochemical systems theory: a review. *ISRN Biomathematics* **2013**, 1–53 (2013). `https://doi.org/10.1155/2013/897658`

[7] Voit, E. O. Modelling metabolic networks using power-laws and S-systems. *Essays in Biochemistry* **45**, 29–40 (2008). doi:10.1042/BSE0450029.

[8] Alves, R., Antunes, F. & Salvador, A. Tools for kinetic modeling of biochemical networks. *Nature Biotechnology* **24**, 667–672 (2006). doi:10.1038/nbt0606-667.

[9] Alves, R., Vilaprinyó, E., Hernández-Bermejo, B. & Sorribas, A. Mathematical formalisms based on approximated kinetic representations for modeling genetic and metabolic pathways. *Biotechnology and Genetic Engineering Reviews* **25**, 1–40 (2008). doi:10.5661/bger-25-1.

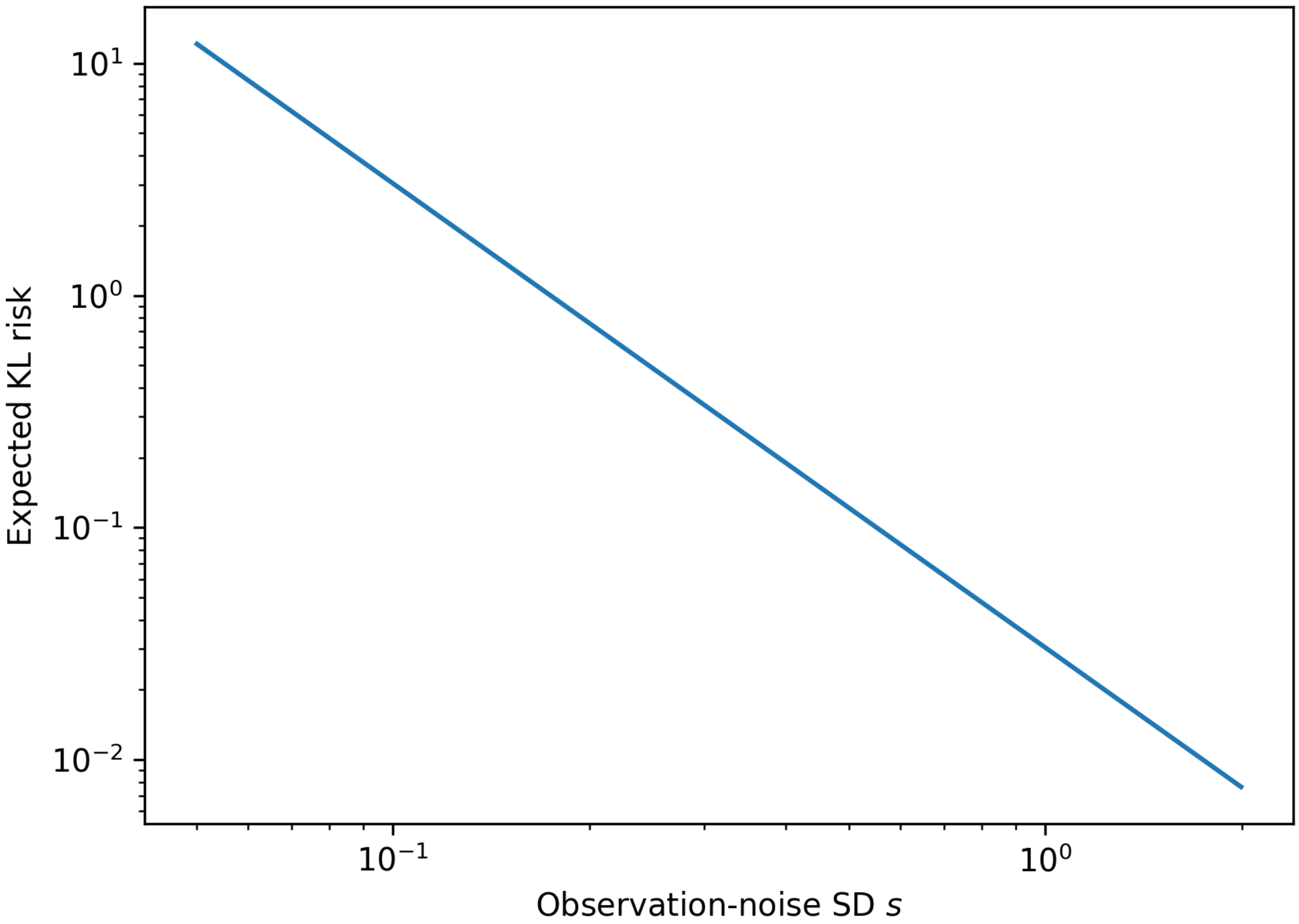

Figure S3: Noise-level dependence. For fixed (H, Σ), both the closed form and Monte Carlo estimates scale approximately as $1/s^2$, as expected from the Gaussian observation model.

[10] Sorribas, A. & Savageau, M. A. Strategies for representing metabolic pathways within biochemical systems theory: reversible pathways. *Mathematical Biosciences* **94**, 239–269 (1989). doi:10.1016/0025-5564(89)90066-7.

[11] Salvador, A. Pillars of theoretical biology: "Biochemical systems analysis, I, II and III". *Journal of Theoretical Biology* **576**, 111655 (2024). doi:10.1016/j.jtbi.2023.111655.

[12] Salvador, A. & Savageau, M. A. Synergism analysis of biochemical systems. I. conceptual framework. *Mathematical Biosciences* **163**, 105–129 (2000). `https://doi.org/10.1016/S0025-5564(99)00056-5`

[13] Salvador, A. & Savageau, M. A. Synergism analysis of biochemical systems. II. tensor formulation and treatment of stoichiometric constraints. *Mathematical Biosciences* **163**, 131–158 (2000). `https://doi.org/10.1016/S0025-5564(99)00057-7`

[14] Cover, T. M. & Thomas, J. A. *Elements of Information Theory* 2nd edn. (Wiley, 2006).

[15] Amari, S.-i. & Nagaoka, H. *Methods of Information Geometry* Vol. 191 of *Translations of Mathematical Monographs* (American Mathematical Society and Oxford University Press, 2000).

[16] Amari, S.-i. *Information Geometry and Its Applications* (Springer, 2016). doi:10.1007/978-4-431-55978-8.

[17] Ay, N. Jost, J. Lê, H. V. & Schwachhöfer, L. *Information Geometry* (Springer, 2017). doi:10.1007/978-3-319-56478-4.

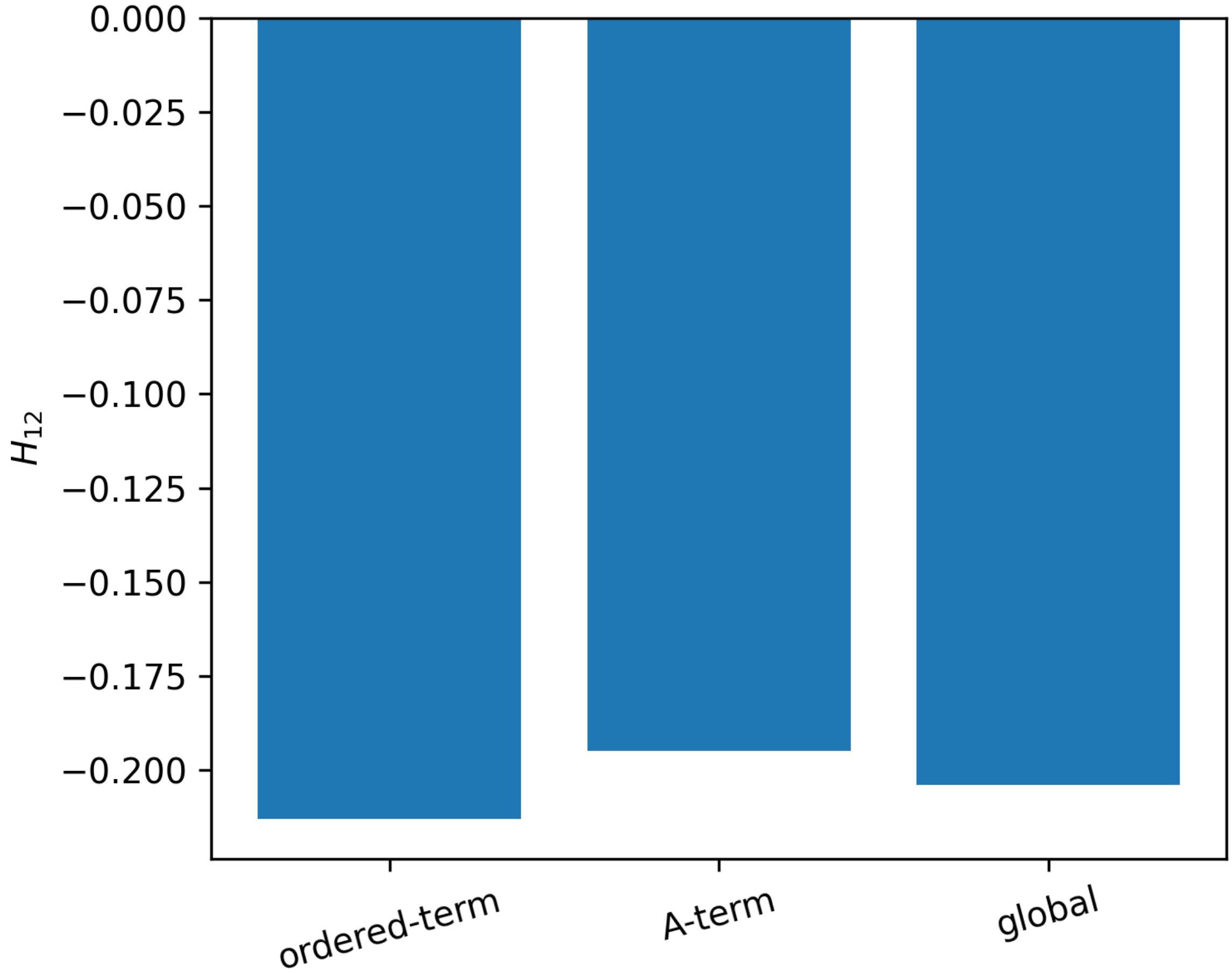

Figure S4: Inhibition mechanism can modify mixed log-curvature and the predicted KL risk. For the ordered Bi–Bi case study, we compare a competitive-inhibition factor applied in the denominator versus a noncompetitive inhibition factor applied to $V_{\max}$ at the same operating point and inhibitor level. The expected KL risk predicted by Eq. (S17) differs through the resulting Hessian entries (log-synergism), illustrating that mechanistic details can matter for interaction-driven failure of a first-order approximation.

[18] Nielsen, F. An elementary introduction to information geometry. *Entropy* **22**, 1100 (2020). doi:10.3390/e22101100.

[19] Fisher, R. A. Theory of statistical estimation. *Proceedings of the Cambridge Philosophical Society* **22**, 700–725 (1925).

[20] Rao, C. R. Information and the accuracy attainable in the estimation of statistical parameters. *Bulletin of the Calcutta Mathematical Society* **37**, 81–91 (1945).

[21] Cramér, H. *Mathematical Methods of Statistics* (Princeton University Press, 1946).

[22] Kay, S. M. *Fundamentals of Statistical Signal Processing, Volume I: Estimation Theory* (Prentice Hall, 1993).

[23] Oehlert, G. W. A note on the delta method. *The American Statistician* **46**, 27–29 (1992).

[24] Anderson, T. W. *An Introduction to Multivariate Statistical Analysis* 3rd edn. (Wiley, 2003).

[25] Kullback, S. *Information Theory and Statistics* (Wiley, 1959).

[26] Cencov, N. N. *Statistical Decision Rules and Optimal Inference* (American Mathematical Society, 1982).

[27] Kass, R. E. & Vos, P. W. *Geometrical Foundations of Asymptotic Inference* (Wiley, 1997).

[28] Dowson, D. C. & Landau, B. V. The Fréchet distance between multivariate normal distributions. *Journal of Multivariate Analysis* **12**, 450–455 (1982).

[29] Gelbrich, M. On a formula for the L^2 Wasserstein metric between measures on Euclidean and Hilbert spaces. *Mathematische Nachrichten* **147**, 185–203 (1990). doi:10.1002/mana.19901470121.

[30] Villani, C. *Optimal Transport: Old and New* (Springer, 2009).

[31] Santambrogio, F. *Optimal Transport for Applied Mathematicians: Calculus of Variations, PDEs and Modeling* (Birkhäuser, 2015). doi:10.1007/978-3-319-20828-2.

[32] Peyré, G. & Cuturi, M. Computational optimal transport. *Foundations and Trends in Machine Learning* **11**, 355–607 (2019). doi:10.1561/2200000073.

[33] Isserlis, L. On a formula for the product-moment coefficient of any order of a normal frequency distribution in any number of variables. *Biometrika* **12**, 134–139 (1918). doi:10.1093/biomet/12.1-2.134.

[34] Mathai, A. M. & Provost, S. B. *Quadratic Forms in Random Variables: Theory and Applications*. Marcel Dekker, New York (1992).

[35] Magnus, J. R. & Neudecker, H. *Matrix Differential Calculus with Applications in Statistics and Econometrics* 3rd edn. Wiley (2019).

[36] Kolda, T. G. & Bader, B. W. Tensor decompositions and applications. *SIAM Review* **51**, 455–500 (2009). doi:10.1137/07070111X.

[37] Kacser, H. & Burns, J. A. in *The control of flux* , Vol. 27 of *Symposia of the Society for Experimental Biology* 65–104 (Cambridge University Press, Cambridge, 1973).

[38] Heinrich, R. & Rapoport, T. A. A linear steady-state treatment of enzymatic chains. general properties, control and effector strength. *European Journal of Biochemistry* **42**, 89–95 (1974).

[39] Fell, D. A. Metabolic control analysis: a survey of its theoretical and experimental development. *Biochemical Journal* **286**, 313–330 (1992).

[40] Hofmeyr, J.-H. S. & Cornish-Bowden, A. Regulating the cellular economy of supply and demand. *FEBS Letters* **476**, 47–51 (2000).

[41] Reder, C. Metabolic control theory: a structural approach. *Journal of Theoretical Biology* **135**, 175–201 (1988).

[42] Kawai, R., Parrondo, J. M. R. & Van den Broeck, C. Dissipation: The phase-space perspective. *Physical Review Letters* **98**, 080602 (2007). `https://doi.org/10.1103/PhysRevLett.98.080602`

[43] Parrondo, J. M. R., Horowitz, J. M. & Sagawa, T. Thermodynamics of information. *Nature Physics* **11**, 131–139 (2015). `https://doi.org/10.1038/nphys3230`

[44] Roldán, É. & Parrondo, J. M. R. Entropy production and Kullback–Leibler divergence between stationary trajectories of discrete systems. *Physical Review E* **85**, 031129 (2012). https://doi.org/10.1103/PhysRevE.85.031129

[45] Battle, C. *et al.* Broken detailed balance at mesoscopic scales in active biological systems. *Science* **352**, 604–607 (2016). https://doi.org/10.1126/science.aac8167

[46] Pickup, L. C. *et al.* Seeing the arrow of time. In *Proceedings of the IEEE Conference on Computer Vision and Pattern Recognition (CVPR)*, 2035–2042 (2014).

[47] Esposito, M. Stochastic entropy production under coarse graining. *Physical Review E* **85**, 041125 (2012).

[48] Still, S., Sivak, D. A., Bell, A. J. & Crooks, G. E. Thermodynamics of prediction. *Physical Review Letters* **109**, 120604 (2012).

[49] Ito, S. & Sagawa, T. Information Thermodynamics on Causal Networks. *Physical Review Letters* **111**, 180603 (2013). doi:10.1103/PhysRevLett.111.180603.

[50] Ito, S. Oizumi, M. & Amari, S.-i. Unified framework for the entropy production and the stochastic interaction based on information geometry. *Physical Review Research* **2**, 033048 (2020). doi:10.1103/PhysRevResearch.2.033048.

[51] Ito, S. & Dechant, A. Stochastic Time Evolution, Information Geometry, and the Cramér–Rao Bound. *Physical Review X* **10**, 021056 (2020). doi:10.1103/PhysRevX.10.021056.

[52] Yoshimura, K. & Ito, S. Information Geometric Inequalities of Chemical Thermodynamics. *Physical Review Research* **3**, 013175 (2021). doi:10.1103/PhysRevResearch.3.013175.

[53] Steuer, R., Gross, T., Selbig, J. & Blasius, B. Structural kinetic modeling of metabolic networks. *Proceedings of the National Academy of Sciences USA* **103**, 11868–11873 (2006). https://doi.org/10.1073/pnas.0600013103

[54] Bruggeman, F. J., Westerhoff, H. V., Hoek, J. B. & Kholodenko, B. N. Modular response analysis of cellular regulatory networks. *Journal of Theoretical Biology* **218**, 507–520 (2002). https://doi.org/10.1006/jtbi.2002.3096
